# Development of Binder-Free Three-Dimensional Honeycomb-like Porous Ternary Layered Double Hydroxide-Embedded MXene Sheets for Bi-Functional Overall Water Splitting Reactions

**DOI:** 10.3390/nano12162886

**Published:** 2022-08-22

**Authors:** Sajjad Hussain, Dhanasekaran Vikraman, Ghazanfar Nazir, Muhammad Taqi Mehran, Faisal Shahzad, Khalid Mujasam Batoo, Hyun-Seok Kim, Jongwan Jung

**Affiliations:** 1 Hybrid Materials Center (HMC), Sejong University, Seoul 05006, Korea; 2 Department of Nanotechnology and Advanced Materials Engineering, Sejong University, Seoul 05006, Korea; 3 Division of Electronics and Electrical Engineering, Dongguk University-Seoul, Seoul 04620, Korea; 4 School of Chemical and Materials Engineering (SCME), National University of Sciences & Technology (NUST), Islamabad 44000, Pakistan; 5 Department of Metallurgy and Materials Engineering, Pakistan Institute of Engineering and Applied Sciences (PIEAS), Islamabad 45650, Pakistan; 6 King Abdullah Institute for Nanotechnology, King Saud University, Riyadh 11451, Saudi Arabia

**Keywords:** LDH, water splitting, HER, OER, MXene

## Abstract

In this study, a honeycomb-like porous-structured nickel–iron–cobalt layered double hydroxide/Ti_3_C_2_T_x_ (NiFeCo–LDH@MXene) composite was successfully fabricated on a three-dimensional nickel foam using a simple hydrothermal approach. Owing to their distinguishable characteristics, the fabricated honeycomb porous-structured NiFeCo–LDH@MXene composites exhibited outstanding bifunctional electrocatalytic activity for pair hydrogen and oxygen evolution reactions in alkaline medium. The developed NiFeCo–LDH@MXene electrocatalyst required low overpotentials of 130 and 34 mV to attain a current density of 10 mA cm^−2^ for OER and HER, respectively. Furthermore, an assembled NiFeCo–LDH@MXene‖NiFeCo–LDH@MXene device exhibited a cell voltage of 1.41 V for overall water splitting with a robust firmness for over 24 h to reach 10 mA cm^−2^ current density, signifying outstanding performance for water splitting reactions. These results demonstrated the promising potential of the designed 3D porous NiFeCo–LDH@MXene sheets as outstanding candidates to replace future green energy conversion devices.

## 1. Introduction

The enormous utility of fossil fuels has appeared as the foremost source of the improvement of poisonous ground-level ozone and airborne matter, which has attracted global concerns [1,2]. Hydrogen, a clean, abundant, renewable, and pollution-free source and sustainable energy carrier element on earth, is considered as an alternative to non-renewable and toxic fossil fuels [3,4,5]. Hydrogen exhibits numerous attractive properties as an energy transferor and a high energy density of 140 MJ/kg, which is three times higher than those of solid fuels [6]. Electrocatalytic water splitting is an easy method to produce high-purity hydrogen [4,7]. Typically, electrocatalytic splitting strategies are driven by two reactions: oxygen evolution reactions (OER) and hydrogen evolution reactions (HER) [8,9,10]. Pt-based materials and IrO_2_/RuO_2_-based metal oxides are considered efficient benchmark catalysts for HER and OER, respectively, owing to their low overpotential, small Tafel slope, and high catalyst activity to facilitate HER/OER processes; however, their natural scarcity, inadequate durability, and high cost have limited their widespread commercial application [11,12]. Therefore, remarkable efforts have been dedicated to develop and improve earth-copious high-enactment catalysts for OER and HER.

The two dimensional (2D) materials offer attractive electronic properties, high mechanical characteristics, superior conductivity, and enormous specific surface area, which supports the promotion of electrocatalysis [13,14]. Recently, the use of numerous electrocatalysts from the layered transition metal chalcogenides (TMDs), carbides (TMC), phosphides, and nitrides to reduce the overpotential of HER has attracted significant research attention owing to their intriguing properties, such as cost, adjustable bandgap alignment, suitable layer spacing, intrinsic behavior, and environmental properties [15,16,17,18]. The most feasible electrocatalyst candidates for OER include metal oxides, layered double hydroxides (LDHs), metal organic framework (MOF) structures, and various carbon-based derivatives [19,20,21]. Among these candidates, LDHs are electrocatalysts with a crystal lamellar organization containing different interlayer anions, metal cations, water molecules, and hydroxyl groups [22,23]. The electrocatalysis of oxygen involves a multi-step redox process, wherein the fundamental process with a maximum overpotential regulates the total turnover frequency [24,25]. However, experimental outcomes have demonstrated the occurrence of electrocatalysis oxygen redox process on the oxide surfaces. LDHs contain earth-abundant elements and are suitable ion/current collectors, environmentally friendly, and exhibit superior stability [26]. Moreover, owing to their distinctive lamellae structure and the interrelated connection among the constituent binary metal, LDH exhibits significant potential for improving electrocatalytic performance [22]. However, binary metal LDHs exhibit a sluggish HER enactment in alkaline solution because of their deficient active edges and meager conductance for HER, which results in large overpotentials and poor kinetics [27]. Various methodologies have been industrialized to increase the HER behavior of LDH, such as development of LDH using trivalent cation and the effective design of composites to enhance the specific area, activate the active edge, and improve the conductance of LDH [22]. Among these approaches, the use of mixed-metal cations containing Fe, Co, and Ni has attracted widespread attention as one of the most proficient electrocatalysts for OER/HER in aqueous environments [28,29,30]. For example, Yao et al. [29] recently developed an efficient CoNiFe–LDH for effective overall water splitting reactions. However, the fabrication of trivalent LDH-containing composites to enhance electrochemical conductivity and generate the numerous catalytic edges to facilitate charge transfer during the HER/OER process has been challenging [24,28,31].

MXenes, a member of a 2D group with a combination of metal carbides and nitrides from the MAX phase, exhibit outstanding properties including, outstanding electrical conductivity, superb intercalation characteristics, and large interlayer spacing with an easily tunable structure composition [32,33]. MXenes exist naturally in semiconductors, semimetals, or superconductors depending on the surface termination or composition. Moreover, they exhibit flexible and superior mechanical properties compared to previously reported 2D materials and exhibit satisfactory operability and easy solubility in any solvent [34,35]. MXenes exhibit enhanced catalytic activity owing to their tuned operative basal edges with exposed metal faces, hydrophilic surface presence with numerous functional groups (–O, –F, –OH, and –Cl), large surface area, and rich porous structure [36,37]. However, designed MXene sheets exhibit poor properties owing to flake/surface terminations that occur during delamination and etching, defective structure, aggregation and restacking of nanosheets, and easy oxidation [38]. Hence, to fully utilize the aforementioned advantages, 2D MXenes can be hybridized with various materials to arrange MXene-based composites to enhance its electrochemical and physical performances. Recently, transition metal oxides, TMDs, polymers, and carbon nanomaterials have been exploited as insertions to avoid the restacking/aggregation and surface terminations of MXene for MXene-based nanocomposites [39,40]. Moreover, to develop inexpensive and effective electrocatalysts for OER/HER, MXene could act as a conductive platform to increase the evolution kinetics of LDH, whereas LDH prevents the restacking of the composite and maintains material stability. To date, various studies have reported the effective water splitting potential of MXene–LDH-based composites, such as CoFe–LDH/MXene [41] FeNi–LDH/Ti_3_C_2_-MXene nanohybrids [42], ripple-like ternary sulfides (sNiFeCo/NF) [43], and MXene/TiO_2_/NiFeCo LDH composites [44].

In this study, we fabricated NiFeCo–LDH@MXene on a 3D nickel foam (NF) to realize an efficient HER/OER catalytic activity through the synthesis of LDH nanostructure-stacked MXene interspace nanosheets on a superiorly interconnected 3D conductive network using an in situ grown hydrothermal reaction. The resulting surface characteristics of the composite verified the in situ self-assembly of the NiFeCo–LDH nanoparticle on the well interconnected porous MXene network. Further, the electrocatalytic activity of the fabricated NiFeCo–LDH@MXene heterostructure for OER and HER was investigated, and the results revealed that the heterostructure required a miniature overpotential of 130 and 34 mV to attain a current density of 10 mA cm^−2^ and Tafel slopes of 52 and 62 mV dec^−1^, respectively, in alkaline medium, which is relatively superior to NiFe–LDH@MXene as well as those of pure MXene and NiFe–LDH. Furthermore, a fabricated NiFeCo–LDH@MXene‖NiFeCo–LDH@MXene device exhibited a cell voltage of 1.41 V for overall water splitting with a robust firmness for over 24 h to realize a 10 mA cm^−2^ current density. The solid interface electrical connections and superior electronic pairing between the two constituents not only reduced the contact resistance but also accelerated electron/ion transport within the NiCoFe–LDH@MXene.

## 2. Materials and Methods

### 2.1. Synthesis of NiFeCo–LDH@MXene Composite

MXene (Ti_3_C_2_T_x_) was extracted from pure MAX (Ti_3_AlC_2_) phase by the perceptive etching of the Al layer engaging hydrogen fluoride (HF) acid, as reported in our previous study [38,45]. To assemble the NiFe–LDHs, FeCl_3_·6H_2_O (0.25 mmol), NiCl_2_·6H_2_O (0.75 mmol), and NH_4_F (2.5 mmol) were added into deionized (DI) water (100 mL), and the solution was blended by continuous smooth magnetic agitation. Subsequently, the aliquots were moved to a stainless steel-braced autoclave. Thereafter, sized NF substrates were vertically incorporated to the autoclaved solution periodically, and the solution temperature was maintained for 12 h at 200 °C. Subsequently, the NiFe–LDHs-deposited NF was collected and dried in a vacuum oven. To fabricate the MXene-based composites, first, the as-prepared MXene was suspended in DI and subjected to 20 min ultrasonic vibration for homogeneous dispersion. Subsequently, FeCl_3_·6H_2_O (0.25 mmol), NiCl_2_·6H_2_O (0.75 mmol), and NH_4_F (2.5 mmol) were added sequentially into the homogeneous MXene solution mixture. Thereafter, NiFe–LDH@MXene composites on NF were fabricated using hydrothermal reaction, which was also used to fabricate the NiFeCo–LDH@MXene composites. Briefly, FeCl_3_·6H_2_O (0.25 mmol), NiCl_2_·6H_2_O (0.75 mmol), CoCl_2_·6H_2_O (0.58 mmol), and NH_4_F (2.5 mmol) were added sequentially into the well-dispersed MXene solution to form a homogeneous solution mixture, after which the NiFeCo–LDH@MXene composites on NF was synthesized using the hydrothermal method. The characterization details are described in the supporting section.

### 2.2. Electrochemical Measurements

All the electrochemical experiments were performed by a three-electrode system using PARSTAT (PMC-1000) electrochemical workstation and a 1 M KOH media for HER and OER operation at room temperature. Linear sweep (LSV) polarization measurements were collected once iR rectification at a sweep speed of 10 mV s^−1^. The active material (MXene, NiFe–LDH, NiFe–LDH@MXene, and NiFeCo–LDH@MXene) on NF were used as the working electrode, an Hg/HgO electrode was utilized as the reference electrode, and a graphite rod was applied as the counter electrode in presence of an alkaline electrolyte for OER and HER. The complete water splitting reactions were performed using NiFeCo–LDH@MXene‖NiFeCo–LDH@MXene, NiFe–LDH@MXene‖NiFeLDH@MXene, and Pt–C‖RuO_2_ device structures. The measured potential values were transformed into reversible hydrogen electrode (RHE) using the ensuing calculation: E(RHE)_HgO_ = E(vs. Hg/HgO) + E^0^_(Hg/HgO)_ + 0.0592 × pH. Electrochemical impedance spectroscopy (EIS) measurements were attained at an applied overpotential in the frequency sort of 1 Hz–1 MHz with an amplitude of 5 mV.

## 3. Results and Discussion

### 3.1. Materials Characteristics

**Figure 1:**
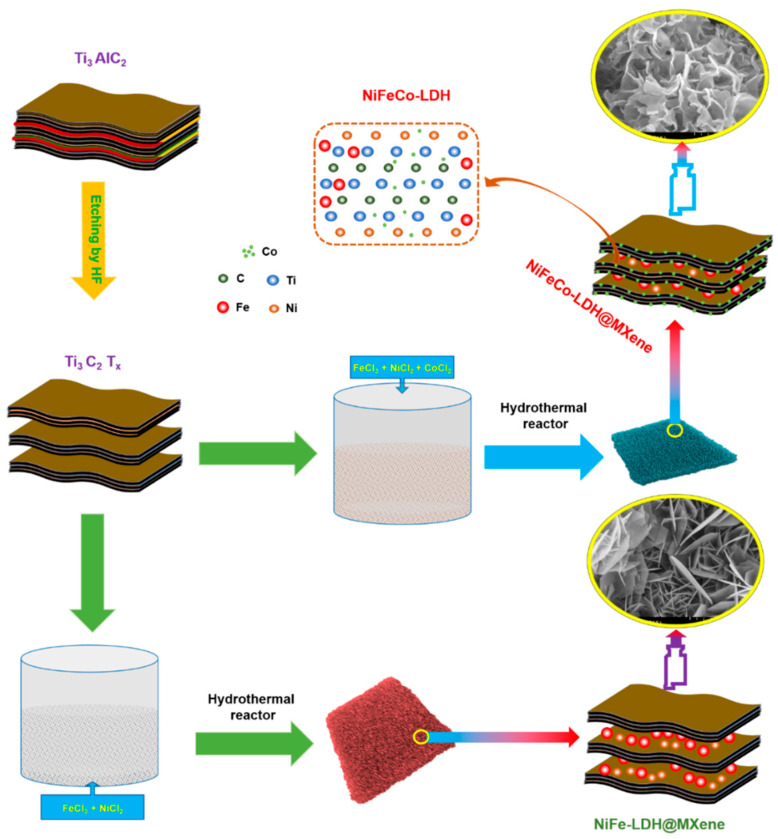
Schematic illustration of the synthetic of mesoporous-structured binary or ternary LDH-decorated MXene composite.

First, MXene sheets were fabricated by a simple HF etching process from the MAX phase Ti_3_AlC_2_, as described in Section 2, and NiFeCo–LDH@MXene and NiFe-LDH@MXene composites were fabricated on 3D NF using a hydrothermal reaction, as shown in Figure 1.

**Figure 2:**
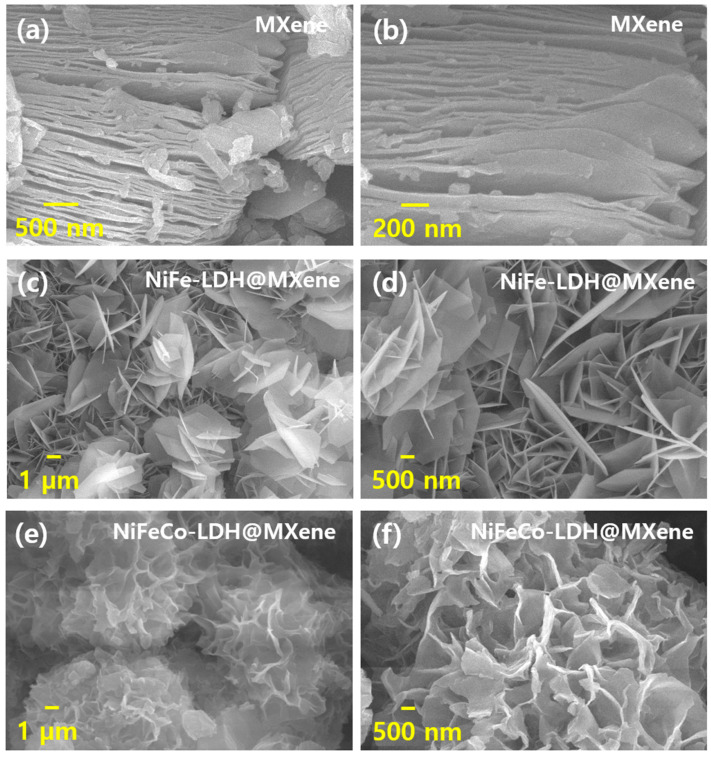
Morphological micrographs of the composites at various magnifications: (**a**,**b**) MXene, (**c**,**d**) NiFe–LDH@MXene, and (**e**,**f**) NiFeCo–LDH@MXene composites.

Figure 2 displays the FESEM images of the synthesized MXene, NiFe–LDH@MXene, and NiFeCo–LDH@MXene nanostructures. The delaminated MXene exhibited a layered sheet-like structure (Figure 2a,b), and the high-magnification micrograph revealed that the etching process resulted in a sequentially-stacked ordered MXene. Figure 2c,d shows the FESEM images of the NiFe–LDH@MXene composites. Homogeneously-dispersed, well-oriented porous-structured micro-leaved grains were largely gathered on the outward of the NiFe–LDH@MXene composite. In addition, the high-magnification image revealed the cross-sectional intersection of the micro-leaves on the porous structures. Figure 2e,f shows the FESEM images of the NiFeCo–LDH@MXene composites. The NiFeCo-LDH@MXene composite exhibited a honeycomb-like morphology, which could be attributed to the inclusion of Co into the NiFe–LDH@MXene matrix. In addition, its high-magnification image confirmed the presence of porous-structured honeycomb grains. Further, to verify the formation of the NiFeCo–LDH@MXene composites, elemental composition and mapping analyses were performed. Figure S1 shows the elemental profile of the NiFeCo–LDH@MXene, which reveals the amalgamated different atom peaks for verifying the composite formation. The elemental profile revealed that the composite was composed of 30, 14.08, 13.7, 22.6, 12.2, and 7.42 at% O, C, Fe, Ni, Co, and Ti, respectively. Figure S2 shows the elemental mapping images of the prepared NiFeCo–LDH@MXene composites. The elemental mapping analysis established the uniform scattering of Ni, Fe, Co, Ti, C, and O elements in the NiFeCo–LDH@MXene composites.

**Figure 3:**
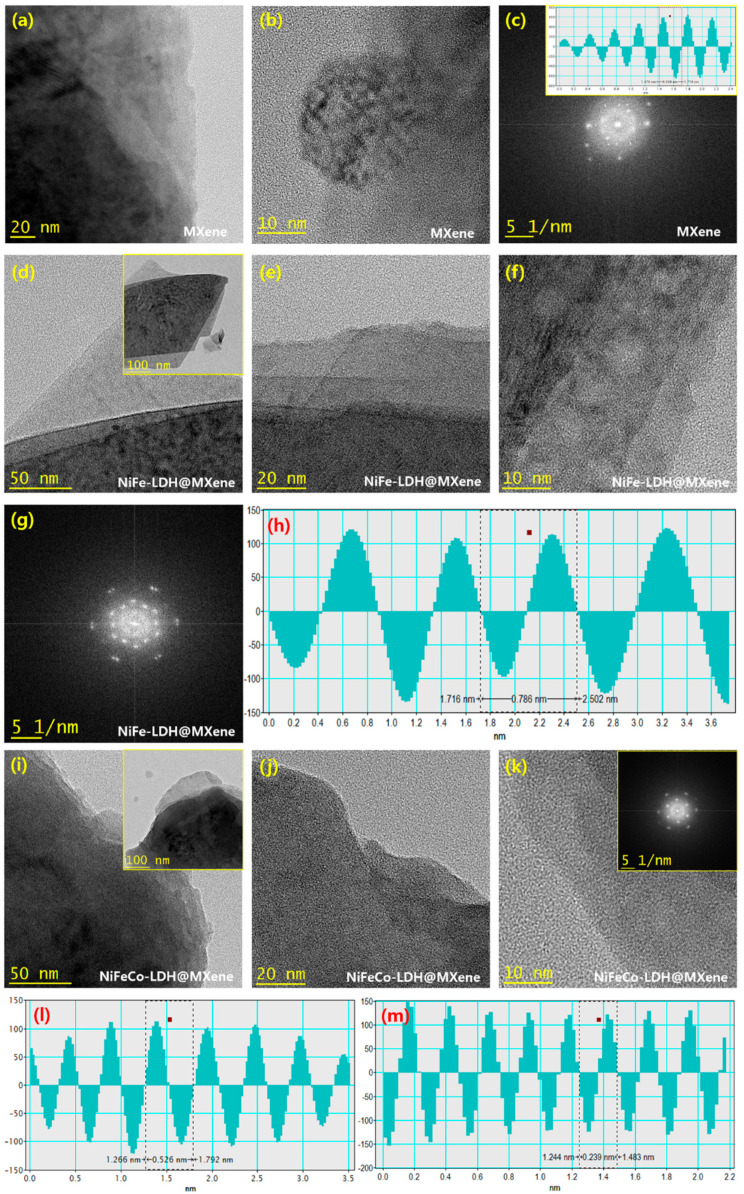
Typical transmission electron microscopy (TEM) imagery of: (**a**–**c**) MXene; (**d**–**h**) NiFe–LDH@MXene composites; and (**i**–**m**) NiFeCo–LDH@MXene composites.

The synthesized MXene, NiFe–LDH@MXene, and NiFeCo–LDH@MXene nanostructures were investigated using TEM. Figure 3a–c shows the TEM images of MXene at different magnifications. Bundles of grain clusters were observed in the TEM image of the sample (Figure 3a). In addition, the surface of the MXene nanostructures was almost completely occupied by disseminated sheets. The fast Fourier transform (FFT) pattern of MXene revealed its polycrystalline crystal direction, which is reliable with the XRD outcomes (Figure 3c). The inset of Figure 3c shows a lattice fringe spacing of 0.338 nm, which parallels the (008) lattice direction of MXene. The TEM images of the NiFe–LDH@MXene composites are shown in Figure 3d–f. Groups of petal-like micro-leaves, which represent dark fringes, were observed in the low-magnification TEM images of NiFe–LDH@MXene (Figure 3d and its inset). In addition, a layered structure and the accumulation of leaf-like grains were detected in the high-magnification imagery (Figure 3e,f). Further, distinctive rod-like grains were perceived in the high-magnification micrographs, which could be attributed to the accumulated leaf structures. Figure 3g shows the FFT profile of the NiFe–LDH@MXene composites. Notable moire fringes with bright doublets were observed in the profile, indicating the formation of LDH polycrystalline crystal. In addition, a lattice arrangement of 0.786 nm was observed in the extracted phase profile (Figure 3h), which corresponded to the (003) lattice direction of LDH, indicating the formation of LDH on the composites. Figure 3i–m shows the TEM images of the NiFeCo–LDH@MXene composite structures. The low-magnification TEM images revealed that the NiFeCo–LDH@MXene composite exhibited a layered architecture under the nanostructured leaf-like grains (Figure 3i and its inset). In addition, a dark mode of porous structures, which was similar to a honey-comb morphology, was observed in the high-magnification images (Figure 3j,k). Further, the extracted FFT profile of the NiFeCo–LDH@MXene composite revealed the presence of moire fringes with ring-patterned bright spots. In addition, a fringe positioning of 0.526 and 0.239 nm was observed in the extracted phase profiles, which could be attributed to the presence of Fe double hydroxide (Figure 3l) and Co double hydroxide (Figure 3m), respectively.

**Figure 4:**
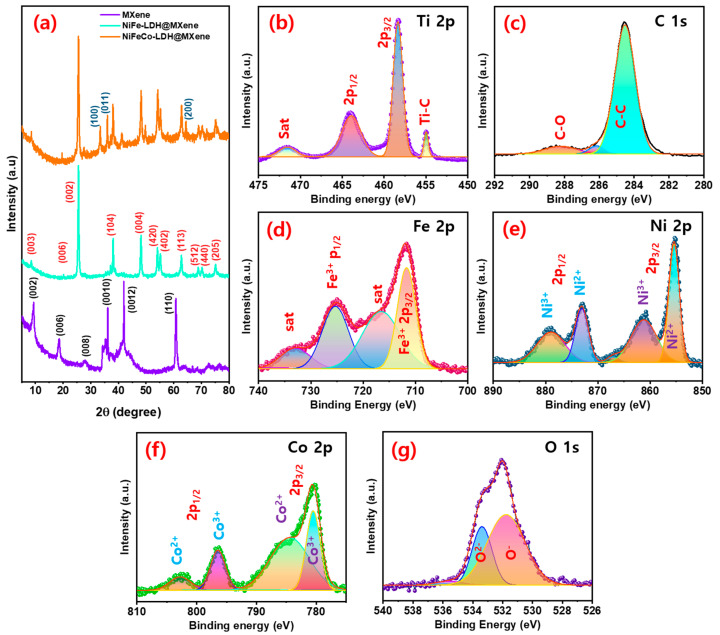
(**a**) X-ray diffraction (XRD) profiles of MXene, NiFe–LDH@MXene, and NiFeCo–LDH@MXene composites; X-ray photoemission spectra of NiFeCo–LDH@MXene composites; (**b**) Ti 2p, (**c**) C 1s, (**d**) Fe 2p, (**e**) Ni 2p, (**f**) Co 2p, and (**g**) O 1s regions.

The structure of the fabricated nanostructures was verified using XRD. Figure S3 shows the XRD profile of the pure MAX phase Ti_3_AlC_2_ MXene. Peaks corresponding to (002), (004), (100), (101), (102), (103), (008), (104), (105), (106), (107), (108), (109), (110), and (1011) lattice planes were observed in the XRD pattern of the pure phase [46]. Further, peaks corresponding to the (002), (006), (008), (0010), (0012), and (110) orientations were observed in the XRD patterns of the delaminated MXene sheets, which are highly consistent with the findings of previous studies (Figure 4a) [47,48]. In addition, crystal planes of (003), (006), (002), (104), (004), (420), (402), (113), (512), (440), and (205), which are also detected in the XRD pattern of NiFe LDH alloys (JCPDS: 89-7111 & 81-2022), as well as low-intensity MXene-related peaks, which could be ascribed to the surface coverage of hydroxide layer, were perceived in the XRD profile of the NiFe–LDH@MXene composite. The combined peaks of NiFe–LDH@MXene and novel peaks of (100), (011), and (200) owing to the presence of Co in the resulting composite (JCPDS: 89-8616) were detected in the XRD profile of the NiFeCo@MXene composites. Moreover, the observed low diffraction angle peak confirmed the layered structure of the composite. These results verified the blending of the strongly-established LDH structure with MXene structure and were consistent with the reports of various reported literatures [49].

The oxidation state and composition of the honeycomb-structured NiFeCo–LDH@MXene composites were corroborated by XPS. The XPS survey scan band of the NiFeCo–LDH@MXene composite discovered the occurrence of Co, Ni, Fe, Ti, C, and O elements in the composite (Figure S4). Figure 4b shows the XPS Ti 2p spectrum containing Ti–C, Ti 2p_3/2_, Ti 2p_1/2_, and TiO_2_-related satellite (sat) bonds [45,50]. The observed Ti–C and sat peaks could be attributed to the realization of diversified carboxides (TiC_x_O_y_) and oxides (TiO_x_F_y_) for the arrangement of MXene [47,51]. In addition, peaks conforming to C–O and sp^2^ C–C bonds in MXene were observed in the C 1s profile of the composite (Figure 4c). Figure 4d shows the Fe 2p region XPS profile of the NiFeCo–LDH@MXene composite. Peaks related to Fe^3+^ and sat peaks related 2p_3/2_ and 2p_1/2_ state, which enabled the mixed bonding of metal alloy and hydroxide in the NiFeCo–LDH@MXene composite, were observed in the XPS profile of the composite [52]. Furthermore, peaks at 853.8 eV (Ni^2+^ 2p_3/2_) and 871.1 eV (Ni^2+^ 2p_1/2_), and two sat (Ni^3+^) peaks at 861.3 (2p_3/2_) and 877.2 eV (2p_1/2_) were observed in the core-level Ni 2p XPS spectrum, indicating the presence of the metallic state and hydroxide form of nickel (Figure 4e). Additionally, the bands conforming to the Co 2p_3/2_ and Co 2p_1/2_ states were observed at 780.1 and 796.2 eV, respectively, in the Co 2p core level spectrum of NiFeCo–LDH@MXene (Figure 4f). The sat peaks observed at 785.8 and 803.6 eV were assigned to the Co(II) state in LDHs [52]. Figure 4g shows the O 1s profile of NiFeCo–LDH@MXene, and peaks corresponding to O^2-^ and O^-^ were observed in the de-convoluted spectrum, which could be attributed to the oxygen defects and lattice oxygen [21]. The XPS results confirmed the formation of NiFeCo–LDH@MXene composites.

Raman analysis was explored to check the vibrational mode of the MXene, NiFe–LDH@MXene, and NiFeCo–LDH@MXene nanostructures as given in Figure S5. For MXene, the characteristic Raman vibrations are at 204, 392, 619, and 722 cm^−1^, along with the graphene related broad and weak D and G bands at 1358 and 1576 cm^−1^, respectively, which concurs with the previous results [38]. For nanocomposites, the peak around 548.3 and 458.8 cm^−1^ are assigned to Fe^3+^−O−Fe^3+^ and Fe^3+^/Ni^2+^−O−Ni^2+^ bonds, which suggested the formation of NiFe-LDH@MXene and NiFeCo-LDH@MXene composites. Moreover, the exhibit of additional peak around 749.3 cm^−1^ represents the Co–Co stretching mode for the NiFeCo-LDH@MXene composites [53]. The well propelled graphitic carbon-related D and G band are exhibited for the LDH nanocomposites, which proved the interactive relations between the MXene and LDHs highly beneficial for improving electrochemical properties [38].

### 3.2. Hydrogen Evolution Reaction

**Figure 5:**
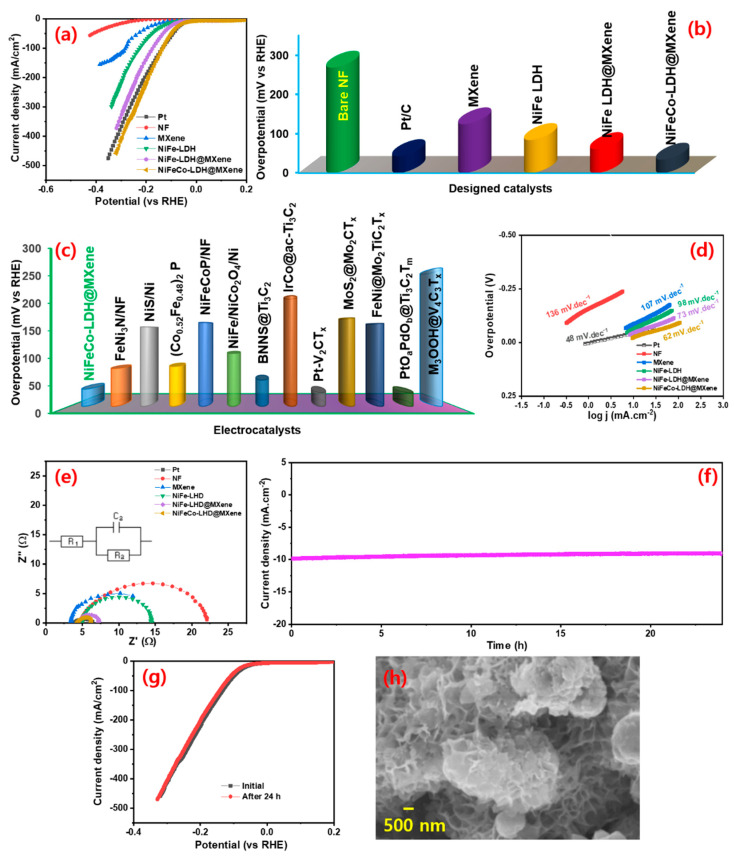
Hydrogen evolution reaction (HER) performance of the electrocatalysts: (**a**) linear sweep voltammetry (LSV) polarizations and (**b**) the HER overpotential variations of bare NF, Pt/C, MXene, NiFe-LDH, NiFe–LDH@MXene, and NiFeCo–LDH@MXene at a scan speed of 10 mV s^−1^; (**c**) comparison of the HER overpotential of NiFeCo–LDH@MXene composite and those of previously reported electrocatalysts; (**d**) Tafel and (**e**) electrochemical impedance spectroscopy (EIS) profiles of bare NF, Pt/C, MXene, NiFe-LDH, NiFe–LDH@MXene, and NiFeCo–LDH@MXene; (**f**) time-dependent current density variations of the NiFeCo–LDH@MXene composite for continuous HER operation for over 24 h at a constant overpotential; (**g**) LSV profiles before and after 24-h continuous HER reaction; (**h**) SEM micrograph of NiFeCo–LDH@MXene composite catalyst after 24 h HER reaction.

The electrochemical HER performance of the assembled (MXene, NiFe-LDH@MXene and NiFeCo-LDH@MXene) electrocatalysts was evaluated in an N_2_-saturated alkaline (1 M KOH) medium. To expand the comparison scope, the electrochemical behavior of noble Pt/C (20 wt%) electrocatalyst and bare NF and NiFe–LDH were also investigated. Figure S6 displays the FESEM imagery of the pure NiFe–LDH. The LSV polarization curves of the samples were recorded at a sweep speed of 10 mV s^−1^ at room temperature. Figure 5a spectacles the LSV profiles of the bare NF, Pt/C, MXene, NiFe–LDH, NiFe–LDH@MXene, and NiFeCo–LDH@MXene electrocatalysts for HER. The profiles indicated that the NiFeCo–LDH@MXene exhibited a noble metal-like behavior. Figure 5b shows the overpotential required by the prepared catalysts to attain a 10 mA cm^−2^ current density. The NiFeCo–LDH@MXene desired a 34 mV vs. RHE of HER overpotential to realize a current density of 10 mA cm^−2^, whereas the noble metal Pt/C required an overpotential of 43 mV vs. RHE to touch the same current density, indicating the superior electrocatalytic activity of NiFeCo–LDH@MXene. In addition, the NF, MXene, NiFe–LDH, and NiFe–LDH@MXene electrocatalysts required HER overpotential of 268, 124, 84, and 61 mV vs. RHE, respectively. Figure 5c shows the comparison of the HER overpotential of the prepared NiFeCo–LDH@MXene and those of various previously reported electrocatalysts, as presented in Table S1.

The HER inherent kinetics in the catalytic process was further examined using Tafel lines. Figure 5d shows the Tafel lines of bare NF, Pt/C, MXene, NiFe–LDH, NiFe–LDH@MXene, and NiFeCo–LDH@MXene electrocatalysts. The Tafel slope values of the bare NF, Pt/C, MXene, NiFe–LDH, NiFe–LDH@MXene, and NiFeCo–LDH@MXene were 136, 48, 107, 98, 73, and 62 mV dec^−1^, respectively. The Tafel slope of the NiFeCo–LDH@MXene composite (62 mV dec^−1^) was less than those of the other designed catalysts, further confirming its enhanced electrocatalytic behavior through the substitution of Co in the NiFe–LDH lattice to form composites. The Co cations modification enhanced the conductivity of the resulting LDH and altered the oxidation state of the surrounding Ni^3+^ or Fe^3+^ sites, thus generating active sites and facilitating swift charge transfer [20]. The formation of binder free 3D microporous NF skeleton interconnected NiFe-LDH@MXene and NiFeCo–LDH@MXene composites greatly enhances the exposure of active site and eliminates the internal resistance caused by use of binder. The low Tafel slope of the NiFeCo–LDH@MXene composite (62 mV dec^−1^) was used to propose the following favorable HER kinetics [54,55].

(1) $$H_{2}O+e^{-}\to H_{\mathrm{ads}}+{\mathrm{OH}}^{-}\;(\mathrm{Discharge}\;\mathrm{or}\;\mathrm{Volmer}\;\mathrm{step})$$

(2) $$H_{\mathrm{ads}}+H_{2}O+{\;e}^{-}\to {\mathrm{OH}}^{-}+{\;H}_{2}\;(\mathrm{Electrochemical}\;\mathrm{desorption}\;\mathrm{or}\;\mathrm{Heyrovsky}\;\mathrm{step})$$

(3) $$H_{\mathrm{ads}}+H_{\mathrm{ads}}\to {\;H}_{2}\;(\mathrm{Chemical}\;\mathrm{combination}\;\mathrm{or}\;\mathrm{Tafel}\;\mathrm{step})$$

where H_ads_ is an adsorbed species. The observed Tafel slope value indicated that the NiFeCo–LDH@MXene composite electrocatalyst conformed to the Volmer–Heyrovsky and/or Volmer–Tafel combined kinetics. Additionally, the exchange current density was appraised by the extrapolation of Tafel lines to the current [7]. The NiFeCo–LDH@MXene electrocatalyst (1.36 mA cm^−2^) exhibited higher estimated exchange current density values than the Pt/C (1.28 mA cm^−2^), bare NF (0.006 mA cm^−2^), MXene (0.08 mA cm^−2^), NiFe-LDH@MXene (0.45 mA cm^−2^), and NiFeCo–LDH (0.09 mA cm^−2^) electrocatalysts. Moreover, the HER characteristics of NiFeCo–LDH@MXene was associated to various described LDH and MXene based electrocatalysts (Table S1).

In this study, EIS was performed to explore the interfacial coupling resistance and current transporting behavior across the catalyst for HER reaction. Figure 5e displays the Nyquist plots with an inset of fitted electrical circuit for the bare NF, Pt/C, MXene, NiFe–LDH, NiFe–LDH@MXene, and NiFeCo–LDH@MXene. A strong interconnection of the hydrothermal grown film on NF was observed in the profiles of the all the catalysts, which could be attributed to their low series resistance (Rs). The exhibited R_S_ values are at 4.8, 3.5, 2.9, 4.9, 4.7, and 4.2 Ω for the bare NF, Pt/C, MXene, NiFe–LDH, NiFe–LDH@MXene, and NiFeCo–LDH@MXene, respectively. In addition, the NiFeCo–LDH@MXene (1.4 Ω) composite exhibited a lower charge transfer resistance (Rct) compared to NiFe–LDH@MXene (3.9 Ω), NiFe (3.9 Ω), and MXene (3.8 Ω), indicating its rapid electron transfer efficiency and high conductivity for HER reaction. The low R_ct_ of the NiFeCo–LDH@MXene composite for HER also indicates a rapid charge transfer kinetic and a high ionic/electronic conductivity by the ternary metal cation-induced MXene layers pathway, which enhanced the conductivity of composite. The HER durability of NiCoFe–LDH@MXene was verified using chronoamperometry measurement for 24 h at a fixed overpotential. Figure 5f shows the time-dependent current variation profile of the NiCoFe–LDH@MXene electrocatalysts for the HER process. A slight decrement in the current density after 24 h HER reaction owing to the formation of hydrogen bubbles and a robust performance was observed. The polarization profiles of the NiCoFe–LDH@MXene catalyst before and after the 24-h HER reaction indicated the persistent stability of the catalyst (Figure 5g). Furthermore, there were no significant changes in the shape of the LSV curves before and after the 24 h continuous HER, implying the robust HER activity of the catalyst for long-term water splitting. Figure 5h shows the SEM micrograph of the catalyst later the 24 h HER operation, which verified the steadiness of prepared NiCoFe–LDH@MXene catalyst.

### 3.3. Oxygen Evolution Reaction

**Figure 6:**
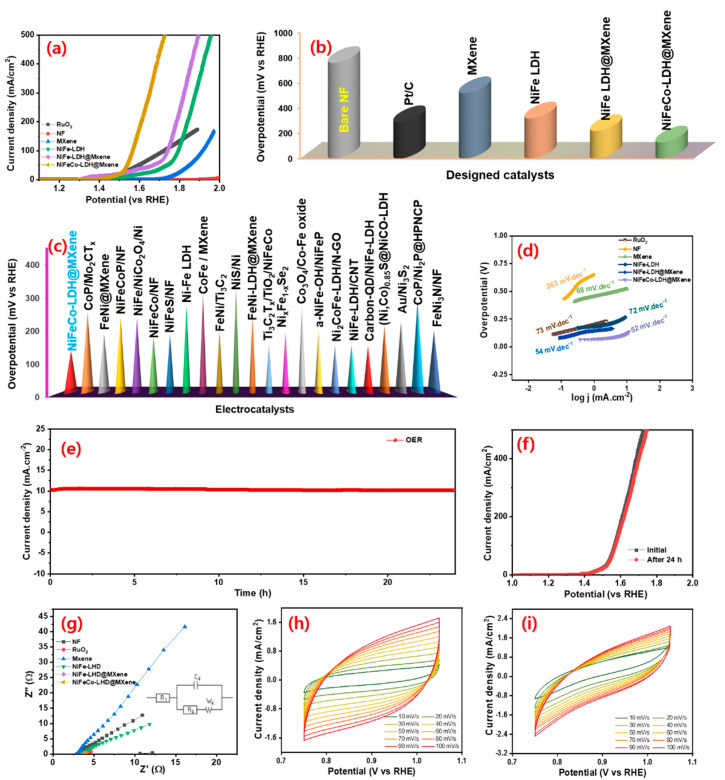
Oxygen evolution reaction (OER) performance: (**a**) LSV polarizations and (**b**) the variations in the OER overpotential of the bare NF, RuO_2_, MXene, NiFe–LDH, NiFe–LDH@MXene, and NiFeCo–LDH@MXene electrocatalysts at a scan speed of 10 mV s^−1^; (**c**) comparison of the OER overpotential of the NiFeCo–LDH@MXene composite to those of previously reported electrocatalysts; (**d**) Tafel profiles of the bare NF, RuO_2_, MXene, NiFe–LDH, NiFe–LDH@MXene, and NiFeCo–LDH@MXene; (**e**) variations in the time-dependent current density of the NiFeCo–LDH@MXene composite for 24 h continuous OER operation at a constant overpotential; (**f**) LSV OER profiles before and after the 24 h continuous OER reaction; (**g**) Nyquist profiles of the bare NF, RuO_2_, MXene, NiFe–LDH, NiFe–LDH@MXene, and NiFeCo–LDH@MXene catalysts; non-faradaic region cyclic voltammetry (CV) profiles of (**h**) NiFe–LDH@MXene and (**i**) NiFeCo–LDH@MXene composite catalysts at different scan rate.

The OER electrocatalytic properties of the designed electrocatalysts were investigated in an alkaline medium. To expand the comparison scope, the electrochemical behavior of a noble OER electrocatalyst (RuO_2_) was also investigated. Figure 6a shows the OER LSV polarization profiles of the bare NF, RuO_2_, MXene, NiFe–LDH, NiFe–LDH@MXene, and NiFeCo–LDH@MXene electrocatalysts. The OER polarization profile demonstrated the significant importance of LDH structure formation for efficient OER kinetics. Figure 6b shows the variation in the overpotential required by the bare NF, RuO_2_, MXene, NiFe–LDH, NiFe–LDH@MXene, and NiFeCo–LDH@MXene to touch a current density of 10 mA cm^−2^. The NiFeCo–LDH@MXene composite required a lower overpotential (∼130 mV vs. RHE) compared to commercial RuO_2_ (~290 mV) to reach a current density of 10 mA cm^−2^. In addition, the bare NF, MXene, NiFe–LDH, and NiFe–LDH@MXene catalysts needed an overpotential of 760, 520, 320, and 220 mV vs. RHE, respectively. These results confirmed the assumption that the high OER catalytic activity of the NiFe–LDH@MXene and NiFeCo–LDH@MXene could be ascribed to the active porous conductive structure, homogeneous incorporation of Co element in NiFe@MXene, ameliorative crystallinity, and the low resistance of the composite to mass transfer between metal cations. Figure 6c shows the comparison of the OER activity of the arranged NiFeCo–LDH@MXene and those of previously reported catalysts as decorated in Table S2. Figure 6d shows the OER Tafel lines of the bare NF, RuO_2_, MXene, NiFe–LDH, NiFe–LDH@MXene, and NiFeCo–LDH@MXene electrocatalysts. The NiFeCo–LDH@MXene composite displayed a narrow Tafel slope of approximately 52 mV dec^−1^, which indicates its outstanding inherent characteristics compared to those of NiFe–LDH@MXene (54 mV dec^−1^), RuO_2_ (73 mV dec^−1^), NiFe–LDH, (72 mV dec^−1^), and MXene (68 mV dec^−1^). The low Tafel slope of the composite could be credited to the efficient hybridization of transition metal LDH (Fe, Ni, and Co) with MXene, which favored faster and proficient OER reaction kinetics.

Further, the OER durability of the NiCoFe–LDH@MXene catalysts was investigated. Figure 6e shows the time-dependent current variations of the NiCoFe–LDH@MXene catalyst under continuous OER process for 24 h at a constant overpotential. The ternary LDH composite electrocatalyst exhibited a robust OER performance. Figure 6f shows the polarization profiles of the catalyst before and after the 24 h OER reaction. There was no significant change in the shape of the LSVs before and after the 24 h OER reaction, implying the robust OER activity of NiCoFe–LDH@MXene. To further confirm the robustness of the designed NiFeCo–LDH@MXene electrocatalysts, XPS measurements were obtained after the 24 h OER reaction and the outcomes are exposed in Figure S7. In addition, the NiFeCo–LDH@MXene OER performance were extensively compared to those of various hitherto stated LDH- and MXene-based electrocatalysts (Table S2). Figure 6g shows the Nyquist plots (inset—fitted circuit) of the bare NF, RuO_2_, MXene, NiFe–LDH, NiFe–LDH@MXene, and NiFeCo–LDH@MXene electrocatalysts at an applied OER overpotential voltage. The exhibited R_S_ values for the OER reaction are at 2.9, 3.9, 2.9, 3.6, 3.6, and 3.8 Ω corresponding to the bare NF, RuO_2_, MXene, NiFe–LDH, NiFe–LDH@MXene, and NiFeCo–LDH@MXene, respectively. Further, the squat Rct of 0.35 Ω observed for NiFeCo–LDH@MXene composite compared to NiFe–LDH@MXene (0.64 Ω), NiFe (0.9 Ω), and MXene (1.2 Ω), signifying the swift electron transfer characteristics for OER reactions. The NiFeCo–LDH@MXene catalyst exhibited a low R_ct_ and small Rs for OER activity. Moreover, to explore the inherent electrochemical surface area (ESA), the double-layer capacitance (C_dl_) was defined by the cyclic voltammograms (CVs) of the catalysts which is described in the supporting information [7,56]. The CV measurement was performed at different sweep speed to examine the electrocatalytic properties of the exposed composite catalysts. Figure 6h,i displays the CV profiles of the NiFe–LDH@MXene and NiFeCo–LDH@MXene in the non-faradaic zone at various sweep speeds. The area of the curves of the both electrocatalysts increased with an increase in the scan speed. Figure S8 shows the change in the current density of the NiFe–LDH@MXene and NiFeCo–LDH@MXene composites at 0.9 V vs. RHE at different sweep rates. NiFeCo–LDH@MXene exhibited a significantly higher C_dl_ (19.7 mF cm^−2^) than NiFe–LDH@MXene (13.8 mF cm^−2^). The ESA values of the catalysts were calculated using the estimated C_dl_ based on a formulated methodology [9]. The ESA values of NiFeCo–LDH@MXene and NiFe–LDH@MXene were 347 and 494 cm^2^, respectively. The petal-like structure of the NiFeCo–LDH decoration on the MXene sheets considerably decreased the resistivity of the composite and exposed more active facets, thus enlightening the electrical conductance of the nanocomposites. The presence of more active enriched sites can promote the strong intimate contact with the electrolyte, and facilitate the transport of electrons and the existence of more faradaic process, thus enhancing the electrochemical characteristics.

### 3.4. Overall Water Splitting

**Figure 7:**
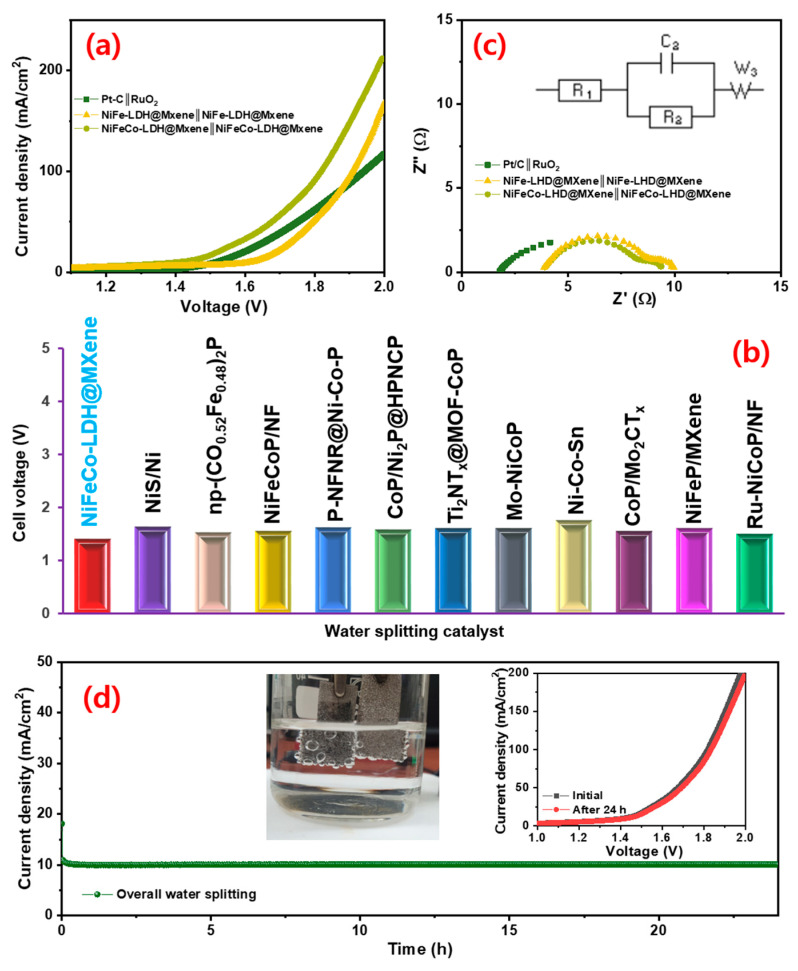
(**a**) Overall water splitting polarization profiles and (**b**) comparison of complete water splitting cell voltage of NiFeCo–LDH@MXene‖NiFe–CoLDH@MXene structures with various LDHs- and MXene-based outcomes; (**c**) EIS profiles of the NiFe–LDH@MXene‖NiFe–LDH@MXene, NiFeCo–LDH@MXene‖NiFe–CoLDH@MXene, and Pt/C‖RuO_2_ devices; (**d**) variations in the time-dependent current density of the NiFeCo–LDH@MXene‖NiFe–CoLDH@MXene device at a constant applied voltage for continuous water splitting operation over 24 h. Inset: two-electrode set up with hydrogen and oxygen bubbles formation; polarization profiles of the device before and after 24 h overall water splitting.

Based on the superior electrocatalytic characteristics of NiFe–LDH@MXene and NiFeCo–LDH@MXene catalysts for both HER and OER, two-electrode NiFe-LDH@MXene (anode)‖NiFe–LDH@MXene (cathode) and NiFeCo-LDH@MXene (anode) ‖NiFe-CoLDH@MXene (cathode) devices were assembled for water overall water splitting reaction at room temperature under alkaline electrolyte. To compare the performance of noble electrocatalyst-based device behavior, the device characteristics of Pt/C‖RuO_2_ was also examined. Figure 7a shows the LSV polarization profiles of the fabricated NiFe–LDH@MXene‖NiFe–LDH@MXene, NiFeCo–LDH@MXene‖NiFe–CoLDH@MXene, and Pt/C‖RuO_2_ cells. The prepared noble NiFeCo–LDH@MXene‖NiFe–CoLDH@MXene device exhibited a low cell voltage of 1.41 V for overall water splitting to realize a current density of 10 mA cm^−2^ at a scan rate of 10 mV s^−1^, which is superior to the other two-electrode alkaline electrolyzer based on NiFe–LDH@MXene‖NiFe–LDH@MXene (1.61 V) and Pt/C‖RuO_2_ (1.75 V). Figure 7b shows the comparison of the cell voltage of the designed NiFe–LDH@MXene device to those of various LDH and MXene-based electrocatalysts for overall water splitting. Figure 7c shows the Nyquist profiles of the assembled electrolyzers at an applied overpotential with an inset of fitted electrical circuit. The observed Rct value of 4.6 Ω (NiFeCo–LDH@MXene) and 5.4 Ω (NiFe–LDH@MXene) assures the intense decay of the charge transfer resistance of NiFeCo–LDH@MXene‖NiFeCo–LDH@MXene, indicating a swift overall water splitting process. Furthermore, the NiFe–LDH@MXene and NiFeCo–LDH@MXene electrolyzer exhibited excellent stability for long-term overall water splitting. Figure S9 and Figure 7d show the chronoamperometric performance of the NiFe–LDH@MXene and NiFeCo–LDH@MXene catalysts at an applied constant corresponding cell voltage for the continuous overall water electrolysis process for over 24 h, respectively. The results demonstrated the robust behavior of the prepared catalyst for overall water splitting without notable degradation at a stable current for over 24 h, confirming the great potential of the catalyst bi-functional water splitting kinetics.

The hierarchical NiFeCo–LDH@MXene hybrid structure as prime electrocatalysts for HER/OER processes exhibited numerous advantages: (i) The unique intrinsic metallic/electronic structure, which enabled a high electronic conductivity of oxide/hydroxide interaction with MXene, significantly enhanced the charge transfer. (ii) MXene functioned as a template to grow the layered NiFeCo–LDH, which significantly enhanced the proficient active edges for high electrocatalytic activity. (iii) The developed hierarchical porous nanosheets acted as “core-shell” arrays structure to facilitate the diffusion of more to access the numerous active sites and make strong intimate contact with electrode, thus promoting ionic/electric diffusion and transport, which enable rapid gas release and a constant working area. (iv) The incorporation of NiFeCo–LDH between the MXene layer enhanced the interlayer spacing, which further enhanced the catalytic activity. (v) The activation of the multi-edges of the 3D network of NF backbones within the MXene and NiFeCo–LDH improved the conductivity of the composite catalyst, thus enhancing its catalytic performance. (vi) The insertion of the NiFeCo–LDH nanosheets hindered the aggregation of MXene layer to enable the full utilization of its entire inherent characteristics, enhance its electrical conductivity and improve the reaction kinetic, thus enhancing the intrinsic catalytic activity of the catalyst.

## 4. Conclusions

This work demonstrated the synthesis of porous-structured NiFeCo–LDHs sheet-embedded MXene composites on a 3D NF network via hydrothermal reaction. The experimental results revealed that the formation of the composites effectively contributed to the tuning of the electronic configuration and surface engineering to efficiently promote the charge/ion diffusion pathways during the HER/OER process and accelerate the reaction kinetics. Electrochemical studies revealed that the fabricated catalyst required small overpotentials of 130 and 34 mV vs. RHE to reach a current density of 10 mA cm^−2^ and Tafel slopes of 62 and 52 mV dec^−1^ for OER and HER, respectively. Furthermore, the NiFeCo–LDH@MXene exhibited prolonged robust performance for over 24 h of continuous and sustained HER/OER owing to their hierarchical structure. Furthermore, an NiFeCo–LDH@MXene‖NiFe-CoLDH@MXene two-electrode device exhibited a low cell voltage of 1.41 V to accomplish a current density of 10 mA cm^−2^ and a robust overall water splitting reaction kinetics for 24 h continuous HER/OER, which exceeded those of the most recently described electrocatalysts and Pt/C–RuO_2_. The outcomes of this study established that the decoration of honeycomb-like porous NiFeCo–LDH@MXene on a 3D network improved the conductivity of the composite and activated multi-active facets for efficient electrocatalytic activity. In addition, the insertion of NiFeCo–LDH nanosheets between the MXene layers effectively reduced the aggregation of MXene layers, thereby enhancing their electrical conductivity and HER/OER reaction kinetics.

## Data Availability

The data presented in this study are available on request from the corresponding author. The data are not publicly available due to ethical.

## Supplementary Materials

The following are available online at: https://www.mdpi.com/article/10.3390/nano12162886/s1, Figure S1: (a,b) SEM- EDX spectrum analysis of NiFeCo-LDH@MXene; (c) elemental wt% their element distribution for Ni, Fe, Co, Ti, and C; Figure S2: (a) FESEM image of NiFeCo-LDH@MXene and (b–f) their elemental mapping images; (b) Ni; (c) Fe; (d); Co; (e) Ti and (f) C elements; Figure S3: XRD pattern of MAX phase Ti3AlC2; Figure S4: XPS survey spectrum of NiFeCo-LDH@MXene; Figure S5: Raman spectrum of MXene, NiFe-LDH@MXene and NiFeCo-LDH@MXene; Figure S6: SEM images of NiFe-LDH (a) low and (b) high magnification; Figure S7: XPS analysis of NiFeCo–LHD@MXene hybrid after 24 h OER performance: (a) survey scan; (b) Fe 2p; (c) Ni 2p; (d) Ti 2p; (e) and (f) Co 2p binding energy; Figure S8: CV profiles current variation at different scan rate at 0.9 V vs. RHE for NiFe-LDH@MXene and NiFeCo-LDH@MXene; Figure S9: Time dependent current density variations of NiFe-LDH@MXene‖NiFe-LDH@MXene device at a constant applied voltage for continuous water splitting operation over 24 h; Table S1: HER catalytic performances LHD-based electrocatalysts; Table S2: OER catalytic performances LHD-based electrocatalysts; Table S3: Comparison of overall water splitting of NiFeCo-LHD@MXene with various electrocatalysts. More detail refer to Refs. [41,42,43,44,57,58,59,60,61,62,63,64,65,66,67,68,69,70,71,72,73,74,75,76,77,78,79,80,81,82,83,84,85,86,87,88,89,90,91,92,93,94].

## References

[1] Li A., Ooka H., Bonnet N., Hayashi T., Sun Y., Jiang Q., Li C., Han H., Nakamura R. (2019). Stable potential windows for long-term electrocatalysis by manganese oxides under acidic conditions. Angew. Chem. Int. Ed..

[2] Gallagher J. (2019). Potential to stabilize. Nat. Energy.

[3] Yan Z., Hitt Jeremy L., Turner John A., Mallouk Thomas E. (2020). Renewable electricity storage using electrolysis. Proc. Natl. Acad. Sci. USA.

[4] Hodges A., Hoang A.L., Tsekouras G., Wagner K., Lee C.-Y., Swiegers G.F., Wallace G.G. (2022). A high-performance capillary-fed electrolysis cell promises more cost-competitive renewable hydrogen. Nat. Commun..

[5] van Renssen S. (2020). The hydrogen solution?. Nat. Clim. Chang..

[6] Beswick R.R., Oliveira A.M., Yan Y. (2021). Does the green hydrogen economy have a water problem?. ACS Energy Lett..

[7] Vikraman D., Hussain S., Rabani I., Feroze A., Ali M., Seo Y.-S., Chun S.-H., Jung J., Kim H.-S. (2021). Engineering mote_2_ and janus semote nanosheet structures: First-principles roadmap and practical uses in hydrogen evolution reactions and symmetric supercapacitors. Nano Energy.

[8] Zhang M., Zhang Y., Ye L., Guo B., Gong Y. (2021). Hierarchically constructed ag nanowires shelled with ultrathin co-ldh nanosheets for advanced oxygen evolution reaction. Appl. Catal. B Environ..

[9] Vikraman D., Hussain S., Karuppasamy K., Feroze A., Kathalingam A., Sanmugam A., Chun S.-H., Jung J., Kim H.-S. (2020). Engineering the novel mose_2_-mo_2_c hybrid nanoarray electrodes for energy storage and water splitting applications. Appl. Catal. B.

[10] Vikraman D., Hussain S., Akbar K., Truong L., Kathalingam A., Chun S.H., Jung J., Park H.J., Kim H.S. (2018). Improved hydrogen evolution reaction performance using mos_2_-ws_2_ heterostructures by physicochemical process. ACS Sustain. Chem. Eng..

[11] Hsieh P.-Y., Wu J.-Y., Chang T.-F.M., Chen C.-Y., Sone M., Hsu Y.-J. (2020). Near infrared-driven photoelectrochemical water splitting: Review and future prospects. Arab. J. Chem..

[12] Yang J., Wang Y., Yang J., Pang Y., Zhu X., Lu Y., Wu Y., Wang J., Chen H., Kou Z. (2022). Quench-induced surface engineering boosts alkaline freshwater and seawater oxygen evolution reaction of porous NiCo_2_O_4_ nanowires. Small.

[13] Tang T., Li S., Sun J., Wang Z., Guan J. (2022). Advances and challenges in two-dimensional materials for oxygen evolution. Nano Res..

[14] Tang T., Wang Z., Guan J. (2022). A review of defect engineering in two-dimensional materials for electrocatalytic hydrogen evolution reaction. Chin. J. Catal..

[15] Lin L., Sherrell P., Liu Y., Lei W., Zhang S., Zhang H., Wallace G.G., Chen J. (2020). Engineered 2D transition metal dichalcogenides—A vision of viable hydrogen evolution reaction catalysis. Adv. Energy Mater..

[16] Zhang H., Yang X., Zhang H., Ma J., Huang Z., Li J., Wang Y. (2021). Transition-metal carbides as hydrogen evolution reduction electrocatalysts: Synthetic methods and optimization strategies. Chem. A Eur. J..

[17] Liu Y., Zhou D., Deng T., He G., Chen A., Sun X., Yang Y., Miao P. (2021). Research progress of oxygen evolution reaction catalysts for electrochemical water splitting. ChemSusChem.

[18] Bai S., Yang M., Jiang J., He X., Zou J., Xiong Z., Liao G., Liu S. (2021). Recent advances of mxenes as electrocatalysts for hydrogen evolution reaction. NPJ 2d Mater. Appl..

[19] Zhou D., Li P., Lin X., McKinley A., Kuang Y., Liu W., Lin W.-F., Sun X., Duan X. (2021). Layered double hydroxide-based electrocatalysts for the oxygen evolution reaction: Identification and tailoring of active sites, and superaerophobic nanoarray electrode assembly. Chem. Soc. Rev..

[20] Wang H.-F., Chen L., Pang H., Kaskel S., Xu Q. (2020). Mof-derived electrocatalysts for oxygen reduction, oxygen evolution and hydrogen evolution reactions. Chem. Soc. Rev..

[21] Bai X., Duan Z., Nan B., Wang L., Tang T., Guan J. (2022). Unveiling the active sites of ultrathin co-fe layered double hydroxides for the oxygen evolution reaction. Chin. J. Catal..

[22] Tang Y., Liu Q., Dong L., Wu H.B., Yu X.-Y. (2020). Activating the hydrogen evolution and overall water splitting performance of NiFe ldh by cation doping and plasma reduction. Appl. Catal. B Environ..

[23] Li C.-F., Xie L.-J., Zhao J.-W., Gu L.-F., Wu J.-Q., Li G.-R. (2022). Interfacial electronic modulation by Fe_2_O_3_/NiFe-LDHS heterostructures for efficient oxygen evolution at high current density. Appl. Catal. B Environ..

[24] Tao H.B., Zhang J., Chen J., Zhang L., Xu Y., Chen J.G., Liu B. (2019). Revealing energetics of surface oxygen redox from kinetic fingerprint in oxygen electrocatalysis. J. Am. Chem. Soc..

[25] Chiu Y.-H., Lai T.-H., Kuo M.-Y., Hsieh P.-Y., Hsu Y.-J. (2019). Photoelectrochemical cells for solar hydrogen production: Challenges and opportunities. APL Mater..

[26] Ping J., Wang Y., Lu Q., Chen B., Chen J., Huang Y., Ma Q., Tan C., Yang J., Cao X. (2016). Self-assembly of single-layer coal-layered double hydroxide nanosheets on 3D graphene network used as highly efficient electrocatalyst for oxygen evolution reaction. Adv. Mater..

[27] Feng X., Jiao Q., Chen W., Dang Y., Dai Z., Suib S.L., Zhang J., Zhao Y., Li H., Feng C. (2021). Cactus-like NiCo_2_S_4_@NiFe LDH hollow spheres as an effective oxygen bifunctional electrocatalyst in alkaline solution. Appl. Catal. B Environ..

[28] Lu X., Sakai N., Tang D., Li X., Taniguchi T., Ma R., Sasaki T. (2020). Conife layered double hydroxide/RuO_2.1_ nanosheet superlattice as carbon-free electrocatalysts for water splitting and Li–O_2_ batteries. ACS Appl. Mater. Interfaces.

[29] Yao J., Xu D., Ma X., Xiao J., Zhang M., Gao H. (2022). Trimetallic conife-layered double hydroxides: Electronic coupling effect and oxygen vacancy for boosting water splitting. J. Power Sources.

[30] Rohit R.C., Jagadale A.D., Lee J., Lee K., Shinde S.K., Kim D.Y. (2021). Tailoring the composition of ternary layered double hydroxides for supercapacitors and electrocatalysis. Energy Fuels.

[31] Li W., Feng B., Yi L., Li J., Hu W. (2021). Highly efficient alkaline water splitting with ru-doped co−v layered double hydroxide nanosheets as a bifunctional electrocatalyst. ChemSusChem.

[32] Gogotsi Y., Anasori B. (2019). The rise of mxenes. ACS Nano.

[33] Bai X., Guan J. (2022). Mxenes for electrocatalysis applications: Modification and hybridization. Chin. J. Catal..

[34] Liu H.-J., Dong B. (2021). Recent advances and prospects of mxene-based materials for electrocatalysis and energy storage. Mater. Today Phys..

[35] Naguib M., Barsoum M.W., Gogotsi Y. (2021). Ten years of progress in the synthesis and development of mxenes. Adv. Mater..

[36] Hussain S., Liu H., Hussain M., Mehran M.T., Kim H.-S., Jung J., Vikraman D., Kang J. (2022). Development of MXene/WO_3_ embedded PEDOT:PSS hole transport layers for highly efficient perovskite solar cells and X-ray detectors. Int. J. Energy Res..

[37] Lipatov A., Lu H., Alhabeb M., Anasori B., Gruverman A., Gogotsi Y., Sinitskii A. (2018). Elastic properties of 2D Ti_3_C_2_T_x_ mxene monolayers and bilayers. Sci. Adv..

[38] Hussain S., Vikraman D., Mehran M.T., Hussain M., Nazir G., Patil S.A., Kim H.-S., Jung J. (2022). Ultrasonically derived WSe_2_ nanostructure embedded mxene hybrid composites for supercapacitors and hydrogen evolution reactions. Renew. Energy.

[39] Kshetri T., Tran D.T., Le H.T., Nguyen D.C., Van Hoa H., Kim N.H., Lee J.H. (2021). Recent advances in mxene-based nanocomposites for electrochemical energy storage applications. Prog. Mater. Sci..

[40] Nan J., Guo X., Xiao J., Li X., Chen W., Wu W., Liu H., Wang Y., Wu M., Wang G. (2021). Nanoengineering of 2D mxene-based materials for energy storage applications. Small.

[41] Hao C., Wu Y., An Y., Cui B., Lin J., Li X., Wang D., Jiang M., Cheng Z., Hu S. (2019). Interface-coupling of CoFe-LDH on mxene as high-performance oxygen evolution catalyst. Mater. Today Energy.

[42] Yu M., Zhou S., Wang Z., Zhao J., Qiu J. (2018). Boosting electrocatalytic oxygen evolution by synergistically coupling layered double hydroxide with mxene. Nano Energy.

[43] Chai Y.-M., Shang X., Liu Z.-Z., Dong B., Han G.-Q., Gao W.-K., Chi J.-Q., Yan K.-L., Liu C.-G. (2018). Ripple-like nifeco sulfides on nickel foam derived from in-situ sulfurization of precursor oxides as efficient anodes for water oxidation. Appl. Surf. Sci..

[44] Hao N., Wei Y., Wang J., Wang Z., Zhu Z., Zhao S., Han M., Huang X. (2018). In situ hybridization of an Mxene/tiO_2_/NiFeCo-layered double hydroxide composite for electrochemical and photoelectrochemical oxygen evolution. RSC Adv..

[45] Hussain S., Rabani I., Vikraman D., Mehran T., Shahzad F., Seo Y.-S., Kim H.-S., Jung J. (2021). Designing the mxene/molybdenum diselenide hybrid nanostructures for high-performance symmetric supercapacitor and hydrogen evolution applications. Int. J. Energy Res..

[46] Li M., Lu J., Luo K., Li Y., Chang K., Chen K., Zhou J., Rosen J., Hultman L., Eklund P. (2019). Element replacement approach by reaction with lewis acidic molten salts to synthesize nanolaminated MAX phases and mxenes. J. Am. Chem. Soc..

[47] Zhu J., Tang Y., Yang C., Wang F., Cao M. (2016). Composites of tio_2_ nanoparticles deposited on ti_3_c_2_ mxene nanosheets with enhanced electrochemical performance. J. Electrochem. Soc..

[48] Tariq A., Ali S.I., Akinwande D., Rizwan S. (2018). Efficient visible-light photocatalysis of 2d-mxene nanohybrids with gd3+-and sn4+-codoped bismuth ferrite. ACS Omega.

[49] Arif M., Yasin G., Shakeel M., Mushtaq M.A., Ye W., Fang X., Ji S., Yan D. (2019). Hierarchical cofe-layered double hydroxide and g-C_3_N_4_ heterostructures with enhanced bifunctional photo/electrocatalytic activity towards overall water splitting. Mater. Chem. Front..

[50] Zhang J., Kong N., Uzun S., Levitt A., Seyedin S., Lynch P.A., Qin S., Han M., Yang W., Liu J. (2020). Scalable manufacturing of free-standing, strong ti3c2tx mxene films with outstanding conductivity. Adv. Mater..

[51] Cao Y., Deng Q., Liu Z., Shen D., Wang T., Huang Q., Du S., Jiang N., Lin C.-T., Yu J. (2017). Enhanced thermal properties of poly(vinylidene fluoride) composites with ultrathin nanosheets of mxene. RSC Adv..

[52] Shabangoli Y., Rahmanifar M.S., El-Kady M.F., Noori A., Mousavi M.F., Kaner R.B. (2018). An integrated electrochemical device based on earth-abundant metals for both energy storage and conversion. Energy Storage Mater..

[53] Sriram B.N., Baby J., Wang S.-F., Ranjitha M.R., Govindasamy M., George M. (2020). Eutectic solvent-mediated synthesis of nife-ldh/sulfur-doped carbon nitride arrays: Investigation of electrocatalytic activity for the dimetridazole sensor in human sustenance. ACS Sustain. Chem. Eng..

[54] Zhang H., Li X., Hähnel A., Naumann V., Lin C., Azimi S., Schweizer S.L., Maijenburg A.W., Wehrspohn R.B. (2018). Bifunctional heterostructure assembly of nife ldh nanosheets on nicop nanowires for highly efficient and stable overall water splitting. Adv. Funct. Mater..

[55] Hussain S., Rabani I., Vikraman D., Feroze A., Karuppasamy K., Haq Z.u., Seo Y.-S., Chun S.-H., Kim H.-S., Jung J. (2020). Hybrid design using carbon nanotubes decorated with Mo_2_C and W_2_C nanoparticles for supercapacitors and hydrogen evolution reactions. ACS Sustain. Chem. Eng..

[56] Hussain S., Rabani I., Vikraman D., Feroze A., Ali M., Seo Y.-S., Song W., An K.-S., Kim H.-S., Chun S.-H. (2021). MoS_2_@X_2_C (x = Mo or W) hybrids for enhanced supercapacitor and hydrogen evolution performances. Chem. Eng. J..

[57] Zhang Y., Gao L., Hensen E.J.M., Hofmann J.P. (2018). Evaluating the stability of co_2_p electrocatalysts in the hydrogen evolution reaction for both acidic and alkaline electrolytes. ACS Energy Lett..

[58] Zhang B., Xiao C., Xie S., Liang J., Chen X., Tang Y. (2016). Iron–nickel nitride nanostructures in situ grown on surface-redox-etching nickel foam: Efficient and ultrasustainable electrocatalysts for overall water splitting. Chem. Mater..

[59] Zhu W., Yue X., Zhang W., Yu S., Zhang Y., Wang J., Wang J. (2016). Nickel sulfide microsphere film on ni foam as an efficient bifunctional electrocatalyst for overall water splitting. Chem. Commun..

[60] Tan Y., Wang H., Liu P., Shen Y., Cheng C., Hirata A., Fujita T., Tang Z., Chen M. (2016). Versatile nanoporous bimetallic phosphides towards electrochemical water splitting. Energy Environ. Sci..

[61] Djire A., Wang X., Xiao C., Nwamba O.C., Mirkin M.V., Neale N.R. (2020). Basal plane hydrogen evolution activity from mixed metal nitride mxenes measured by scanning electrochemical microscopy. Adv. Funct. Mater..

[62] Wang J., He P., Shen Y., Dai L., Li Z., Wu Y., An C. (2021). Feni nanoparticles on mo2tic2tx mxene@ nickel foam as robust electrocatalysts for overall water splitting. Nano Res..

[63] Wang W., Zhao H., Du Y., Yang Y., Li S., Yang B., Liu Y., Wang L. (2021). Rational design and controlled synthesis of v-doped ni3s2/nixpy heterostructured nanosheets for the hydrogen evolution reaction. Chem.-A Eur. J..

[64] Cen J., Wu L., Zeng Y., Ali A., Zhu Y., Shen P.K. (2021). Heterogeneous nifecop/nf nanorods as a bifunctional electrocatalyst for efficient water electrolysis. ChemCatChem.

[65] Cen J., Shen P.K., Zeng Y. (2022). Ru doping nicop hetero-nanowires with modulated electronic structure for efficient overall water splitting. J. Colloid Interface Sci..

[66] Xiao C., Li Y., Lu X., Zhao C. (2016). Bifunctional porous nife/nico2o4/ni foam electrodes with triple hierarchy and double synergies for efficient whole cell water splitting. Adv. Funct. Mater..

[67] Pang Y., Xu W., Zhu S., Cui Z., Liang Y., Li Z., Wu S., Chang C., Luo S. (2021). Self-supporting amorphous nanoporous nifecop electrocatalyst for efficient overall water splitting. J. Mater. Sci. Technol..

[68] Ai Z., Chang B., Xu C., Huang B., Wu Y., Hao X., Shao Y. (2019). Interface engineering in the bnns@ ti 3 c 2 intercalation structure for enhanced electrocatalytic hydrogen evolution. New J. Chem..

[69] Le T.A., Tran N.Q., Hong Y., Kim M., Lee H. (2020). Porosity-engineering of mxene as a support material for a highly efficient electrocatalyst toward overall water splitting. ChemSusChem.

[70] Liu S., Lin Z., Wan R., Liu Y., Liu Z., Zhang S., Zhang X., Tang Z., Lu X., Tian Y. (2021). Cobalt phosphide supported by two-dimensional molybdenum carbide (mxene) for the hydrogen evolution reaction, oxygen evolution reaction, and overall water splitting. J. Mater. Chem. A.

[71] Liu Y., Lu H., Kou X. (2019). Electrodeposited ni-co-sn alloy as a highly efficient electrocatalyst for water splitting. Int. J. Hydrogen Energy.

[72] Park S., Lee Y.-L., Yoon Y., Park S.Y., Yim S., Song W., Myung S., Lee K.-S., Chang H., Lee S.S. (2022). Reducing the high hydrogen binding strength of vanadium carbide mxene with atomic pt confinement for high activity toward her. Appl. Catal. B Environ..

[73] Feng Y., Wang R., Dong P., Wang X., Feng W., Chen J., Cao L., Feng L., He C., Huang J. (2021). Enhanced electrocatalytic activity of nickel cobalt phosphide nanoparticles anchored on porous n-doped fullerene nanorod for efficient overall water splitting. ACS Appl. Mater. Interfaces.

[74] Ren J., Zong H., Sun Y., Gong S., Feng Y., Wang Z., Hu L., Yu K., Zhu Z. (2020). 2d organ-like molybdenum carbide (mxene) coupled with mos 2 nanoflowers enhances the catalytic activity in the hydrogen evolution reaction. CrystEngComm.

[75] Liu H., Cheng J., He W., Li Y., Mao J., Zheng X., Chen C., Cui C., Hao Q. (2022). Interfacial electronic modulation of ni3s2 nanosheet arrays decorated with au nanoparticles boosts overall water splitting. Appl. Catal. B Environ..

[76] Cui B., Hu B., Liu J., Wang M., Song Y., Tian K., Zhang Z., He L. (2018). Solution-plasma-assisted bimetallic oxide alloy nanoparticles of pt and pd embedded within two-dimensional ti3c2t x nanosheets as highly active electrocatalysts for overall water splitting. ACS Appl. Mater. Interfaces.

[77] Du C.F., Sun X., Yu H., Fang W., Jing Y., Wang Y., Li S., Liu X., Yan Q. (2020). V4c3t x mxene: A promising active substrate for reactive surface modification and the enhanced electrocatalytic oxygen evolution activity. InfoMat.

[78] Zhang R., Zhu R., Li Y., Hui Z., Song Y., Cheng Y., Lu J. (2020). Cop and ni 2 p implanted in a hollow porous n-doped carbon polyhedron for ph universal hydrogen evolution reaction and alkaline overall water splitting. Nanoscale.

[79] Zong H., Qi R., Yu K., Zhu Z. (2021). Ultrathin ti2ntx mxene-wrapped mof-derived cop frameworks towards hydrogen evolution and water oxidation. Electrochim. Acta.

[80] Lin J., Yan Y., Li C., Si X., Wang H., Qi J., Cao J., Zhong Z., Fei W., Feng J. (2019). Bifunctional electrocatalysts based on mo-doped nicop nanosheet arrays for overall water splitting. Nano-Micro Lett..

[81] Shen B., Huang H., Jiang Y., Xue Y., He H. (2022). 3D interweaving mxene–graphene network–confined ni–fe layered double hydroxide nanosheets for enhanced hydrogen evolution. Electrochim. Acta.

[82] Dong B., Zhao X., Han G.-Q., Li X., Shang X., Liu Y.-R., Hu W.-H., Chai Y.-M., Zhao H., Liu C.-G. (2016). Two-step synthesis of binary ni–fe sulfides supported on nickel foam as highly efficient electrocatalysts for the oxygen evolution reaction. J. Mater. Chem. A.

[83] Yu L., Yang J.F., Guan B.Y., Lu Y., Lou X.W. (2018). Hierarchical hollow nanoprisms based on ultrathin ni-fe layered double hydroxide nanosheets with enhanced electrocatalytic activity towards oxygen evolution. Angew. Chem. Int. Ed..

[84] Khodabakhshi M., Chen S., Ye T., Wu H., Yang L., Zhang W., Chang H. (2020). Hierarchical highly wrinkled trimetallic nifecu phosphide nanosheets on nanodendrite ni3s2/ni foam as an efficient electrocatalyst for the oxygen evolution reaction. ACS Appl. Mater. Interfaces.

[85] Xu X., Song F., Hu X. (2016). A nickel iron diselenide-derived efficient oxygen-evolution catalyst. Nat. Commun..

[86] Wang X., Yu L., Guan B.Y., Song S., Lou X.W. (2018). Metal–organic framework hybrid-assisted formation of co3o4/co-fe oxide double-shelled nanoboxes for enhanced oxygen evolution. Adv. Mater..

[87] Liang H., Gandi A.N., Xia C., Hedhili M.N., Anjum D.H., Schwingenschlogl U., Alshareef H.N. (2017). Amorphous nife-oh/nifep electrocatalyst fabricated at low temperature for water oxidation applications. ACS Energy Lett..

[88] Zhou D., Cai Z., Lei X., Tian W., Bi Y., Jia Y., Han N., Gao T., Zhang Q., Kuang Y. (2018). Nicofe-layered double hydroxides/n-doped graphene oxide array colloid composite as an efficient bifunctional catalyst for oxygen electrocatalytic reactions. Adv. Energy Mater..

[89] Gong M., Li Y., Wang H., Liang Y., Wu J.Z., Zhou J., Wang J., Regier T., Wei F., Dai H. (2013). An advanced ni–fe layered double hydroxide electrocatalyst for water oxidation. J. Am. Chem. Soc..

[90] Tang D., Liu J., Wu X., Liu R., Han X., Han Y., Huang H., Liu Y., Kang Z. (2014). Carbon quantum dot/nife layered double-hydroxide composite as a highly efficient electrocatalyst for water oxidation. ACS Appl. Mater. Interfaces.

[91] Xia C., Jiang Q., Zhao C., Hedhili M.N., Alshareef H.N. (2016). Selenide-based electrocatalysts and scaffolds for water oxidation applications. Adv. Mater..

[92] Hu L., Li M., Wei X., Wang H., Wu Y., Wen J., Gu W., Zhu C. (2020). Modulating interfacial electronic structure of coni ldh nanosheets with ti3c2tx mxene for enhancing water oxidation catalysis. Chem. Eng. J..

[93] Chen J., Long Q., Xiao K., Ouyang T., Li N., Ye S., Liu Z.-Q. (2021). Vertically-interlaced nifep/mxene electrocatalyst with tunable electronic structure for high-efficiency oxygen evolution reaction. Sci. Bull..

[94] Li Z., Wang X., Ren J., Wang H. (2022). Nife ldh/ti3c2tx/nickel foam as a binder-free electrode with enhanced oxygen evolution reaction performance. Int. J. Hydrogen Energy.

