# Supplementary material for: Development of Binder-Free Three-Dimensional Honeycomb-like Porous Ternary Layered Double Hydroxide-Embedded MXene Sheets for Bi-Functional Overall Water Splitting Reactions
Source: Nanomaterials (Basel). 2022 Aug 22;12(16):2886. doi: 10.3390/nano12162886 (PMC9412967; doi:10.3390/nano12162886)
Supplement: Supplementary file 1 [file nanomaterials-12-02886-s001.zip › nanomaterials-1873765-supplementary.pdf]

nanomaterials

Supporting Information

Development of Binder-Free Three-Dimensional Honeycomb-like Porous Ternary Layered Double Hydroxide-Embedded MXene Sheets for Bi-Functional Overall Water Splitting Reactions

Sajjad Hussain [1,2], Dhanasekaran Vikraman [3], Ghazanfar Nazir [2], Muhammad Taqi Mehran [4], Faisal Shahzad [5], Khalid Mujasam Batoo [6], Hyun-Seok Kim [3] and Jongwan Jung [1,2,*]

[1] Hybrid Materials Center (HMC), Sejong University, Seoul 05006, Korea
[2] Department of Nanotechnology and Advanced Materials Engineering, Sejong University, Seoul 05006, Korea
[3] Division of Electronics and Electrical Engineering, Dongguk University-Seoul, Seoul 04620, Korea
[4] School of Chemical and Materials Engineering (SCME), National University of Sciences & Technology (NUST), Islamabad 44000, Pakistan
[5] Department of Metallurgy and Materials Engineering, Pakistan Institute of Engineering and Applied Sciences (PIEAS), Islamabad 45650, Pakistan
[6] King Abdullah Institute for Nanotechnology, King Saud University, Riyadh 11451, Saudi Arabia
* Correspondence: jwjung@sejong.ac.kr; Tel.: +82-2-3408-3688; Fax: +82-2-3408-4342

Characterizations:

The prepared structures were confirmed using X-ray diffraction (XRD; Rigaku D/max-2500 diffractometer) analysis with Cu-K$_\alpha$ radiance. X-ray photoelectron spectroscopy (XPS) was performed using PHI 5000 Versa Probe (Al Kα radiation source, 25 W, 6.7 × 10^{-8} Pa). The variations in the surface morphology of the prepared nanostructures were validated using transmission electron microscopy (TEM; JEOL-2010F) and X-ray elemental analysis combined with field-emission scanning electron microscopy (FESEM; JEOL JSM-6700F).

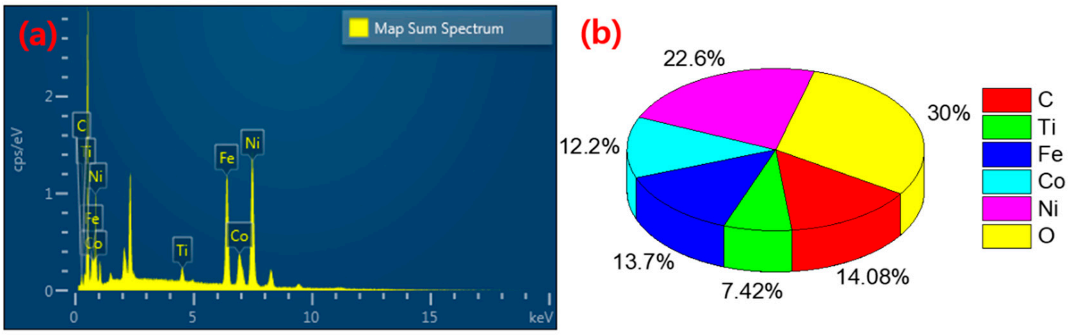

Figure S1. (**a**) SEM- EDX spectrum analysis of NiFeCo-LDH@MXene; (**b**) elemental wt % their element distribution for Ni, Fe, Co, Ti, and C.

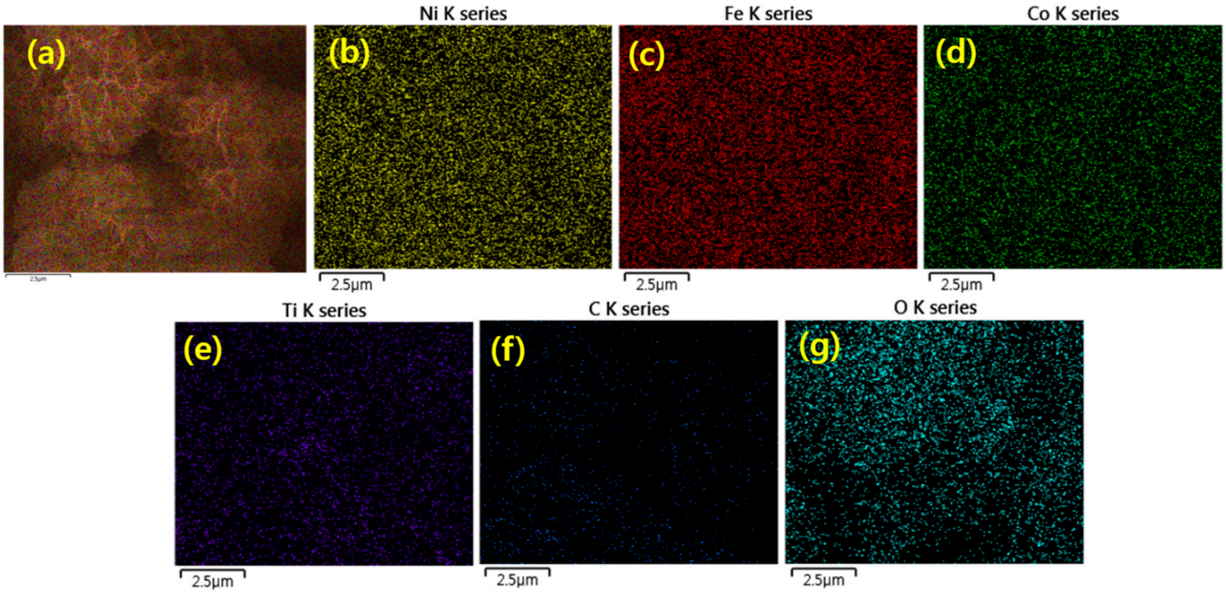

Figure S2. (**a**) FESEM image of NiFeCo-LDH@MXene and (**b**–**f**) their elemental mapping images; (**b**) Ni ; (**c**) Fe ; (**d**) ;Co ; (**e**) Ti and (**f**) C elements.

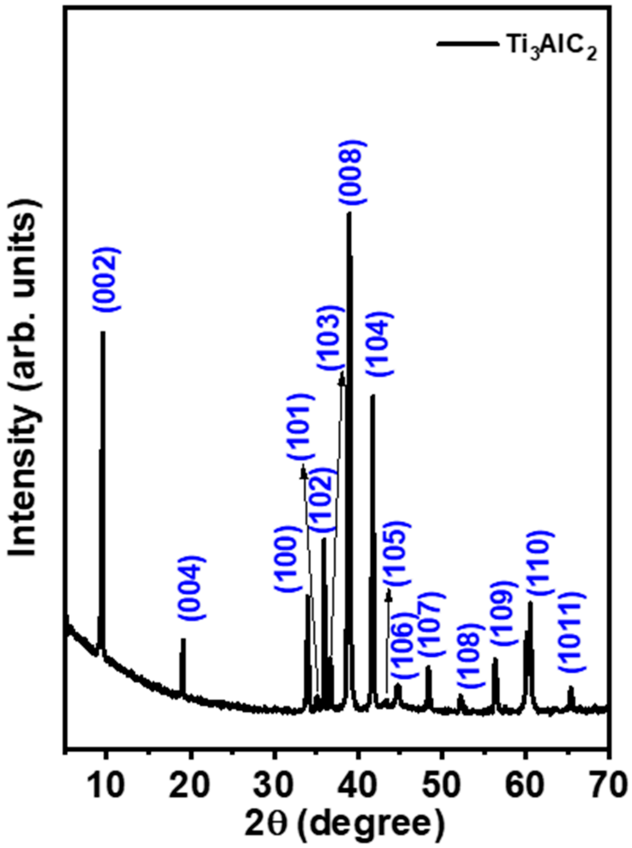

Figure S3. XRD pattern of MAX phase Ti_3AlC_2.

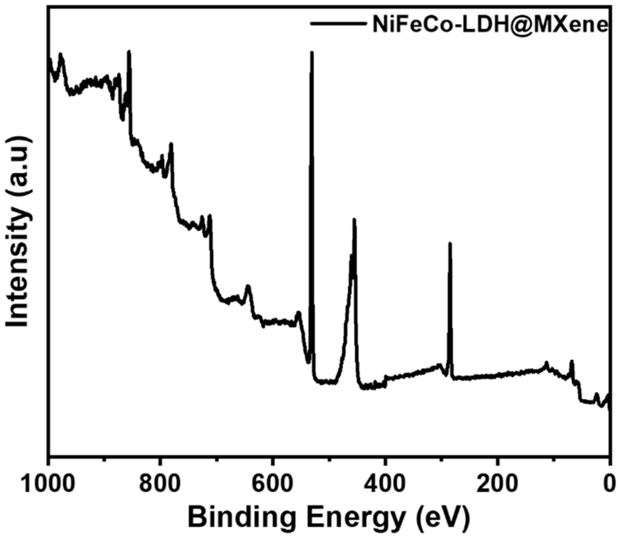

Figure S4. XPS survey spectrum of NiFeCo-LDH@MXene.

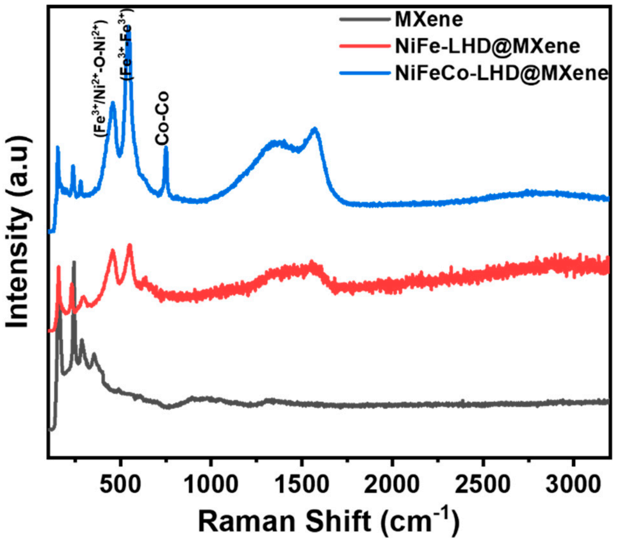

Figure S5. Raman spectrum of MXene, NiFe-LDH@MXene and NiFeCo-LDH@MXene.

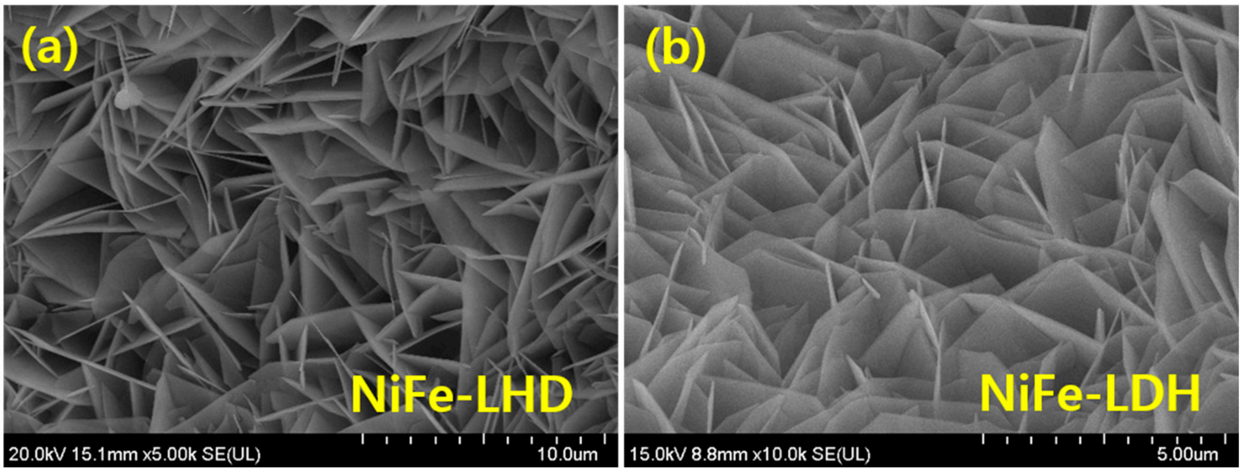

Figure S6. SEM images of NiFe-LDH (**a**) low and (**b**) high magnification.

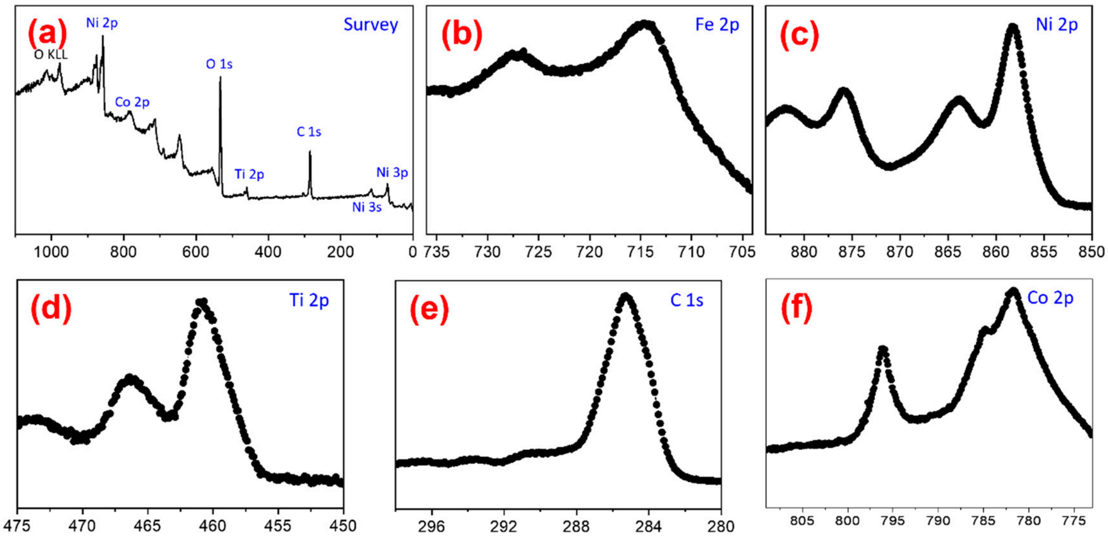

Figure S7. XPS analysis of NiFeCo-LHD@MXene hybrid after 24 h OER performance: (**a**) survey scan; (**b**) Fe 2p; (**c**) Ni 2p; (d) Ti 2p; (**e**) and (**f**) Co 2p binding energy.

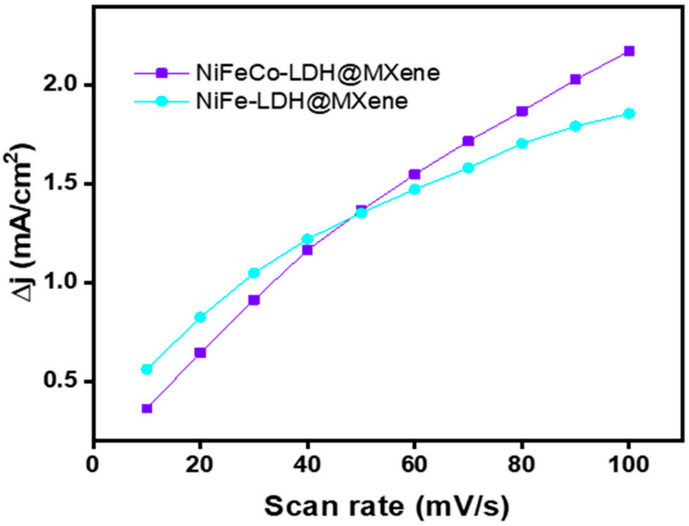

Figure S8. CV profiles current variation at different scan rate at 0.9 V vs RHE for NiFe-LDH@MXene and NiFeCo-LDH@MXene.

ESA calculation:

ESA were evaluated using the following relation:

$$ESA = {}^{C_{dl}}/_{C_s}$$

where, C_{dl} is the double layer capacitance and C_s is the specific capacitance. C_s = 0.040 mF.cm^{-2} for 1 M KOH.[1] C_{dl} values were estimated by the relation $i_d = \nu C_{dl}$, where i_d is the current differences from double layer anodic and cathodic current density, ν is the scan rate and C_{dl} value evaluated from the fitted slope value.

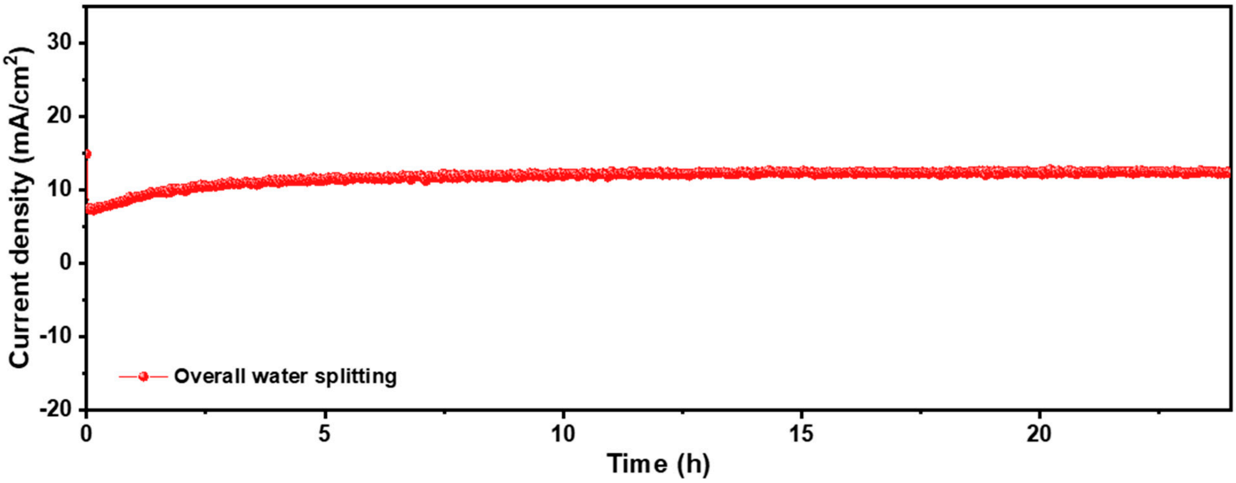

Figure S9. Time dependent current density variations of NiFe-LDH@MXene‖NiFe-LDH@MXene device at a constant applied voltage for continuous water splitting operation over 24 h

Table S1. HER catalytic performances LHD-based electrocatalysts.

Electrocatalyst	Electrolyte	η (mV)	Tafel Slope ($mV \cdot dec^{-1}$)	Ref
NiFeCo-LHD@MXene	1M KOH	34 @ 10 mA/cm^2	62	This work
$FeNi_3N$/NF	1M KOH	75 @ 10 mA/cm^2	98	[2]
NiS/Ni	1M KOH	158 @ 20 mA/cm^2	83	[3]
np-$(Co_{0.52}Fe_{0.48})_2$ P	1M KOH	79 @ 20 mA/cm^2	40	[4]
V-$Ti_4N_3T_x$	0.5 M H_2SO_4	300 @ 10 mA/cm^2	190	[5]
FeNi@MXene ($Mo_2TiC_2T_x$)	1M KOH	160@ 10 mA/cm^2	103.46	[6]
V-Ni_3S_2/Ni_xP_y/NF	1M KOH	90@ 10 mA/cm^2	94.9	[7]
NiFeCoP/NF Nanorod	1M KOH	82.7@10 mA/cm	84.8	[8]
Ru-NiCoP/NF	1M KOH	32.33@ 10 mA/cm^2	60.97	[9]
NiFe/$NiCo_2O_4$/Ni	1M KOH	105@10 mA/cm	88	[10]
np-NiFeCoP	1M KOH	105@10 mA/cm	61.7	[11]
BNNS@Ti_3C_2	0.5M H_2SO_4	52@10 mA/cm	39	[12]
IrCo@ac-Ti_3C_2	1M KOH	220@10 mA/cm	60	[13]
CoP/Mo_2CT_x	1M KOH	78@10 mA/cm	66	[14]
Ni-Co-Sn	1M KOH	76@10 mA/cm	63	[15]
Pt-V2CTx MXene	0.5M H_2SO_4	27@10 mA/cm	36.5	[16]
p-NFNR@Ni-Co-P	1M KOH	125@10 mA/cm	85	[17]
MoS_2@Mo_2CTx	0.5M H_2SO_4	176@10 mA/cm	207	[18]
Au/Ni_3S_2	1M KOH	97@10 mA/cm	72	[19]
FeNi@$Mo_2TiC_2T_x$@NF	0.5M H_2SO_4	165@10 mA/cm	103.46	[6]
PtOaPdOb nanoparticles@$Ti_3C_2T_m$	0.5M H_2SO_4	26.5@10 mA/cm	39	[20]
M_3OOH@$V_4C_3T_x$	1M KOH	275.2@10 mA/cm	51.4	[21]
CoP/Ni_2P@HPNCP	1M KOH	106@10 mA/cm	65.9	[22]
Ti_2NT_x@MOF-CoP	1M KOH	112@10 mA/cm	67.1	[23]
Mo–NiCoP	1M KOH	76@10 mA/cm	60	[24]
NiFe-LDH/MXene-RGO	1M KOH	362@10 mA/cm	100	[25]

Table S2. OER catalytic performances LHD-based electrocatalysts.

Electrocatalyst	Electrolyte	η (mV)	Tafel Slope ($mV·dec^{-1}$)	Ref
NiFeCo-LHD@MXene	1M KOH	130 @ 10 mA/cm^2	52	This work
$FeNi_3N$/NF	1M KOH	202 @ 10 mA/cm^2	40	[2]
NiS/Ni	1M KOH	335 @ 50 mA/cm^2	89	[3]
np-$(Co_{0.52}Fe_{0.48})_2$ P	1M KOH	270 @ 10 mA/cm^2	30	[4]
NiFeCoP/NF Nanorod	1M KOH	244.4@100 mA/cm	40.90	[8]
Ru-NiCoP/NF	1M KOH	233.77@ 50 mA/cm^2	52.66	[9]
NiFe/$NiCo_2O_4$/Ni	1M KOH	240@10 mA/cm^2	38.8	[10]
Ripple-like sNiFeCo/NF	1M KOH	180 @ 100 mA/cm^2	50.4	[26]
FeNi@MXene ($Mo_2TiC_2T_x$)	1M KOH	190@ 10 mA/cm^2	42.78	[6]
np-NiFeCoP	1M KOH	220@10 mA/cm	41.4	[11]
NiFeS/NF	1M KOH	189 @ 100 mA/cm^2	119.4	[27]
CoP/Mo_2CT_x	1M KOH	260@10 mA/cm	51	[14]
Ni-Fe LDH hollow nanoprisms	1M KOH	280 @ 100 mA/cm^2	49.4	[28]
NiFeCoP/NF	1M KOH	109@10 mA/cm	40.90	[8]
CoFe-LDH on MXene	1M KOH	319 @ 10 mA/cm^2	50	[29]
Ni-Co-Sn	1M KOH	270@10 mA/cm	62	[15]
Au/Ni_3S_2	1M KOH	230@10 mA/cm	51	[19]
FeNi-LDH/Ti_3C_2-MXene	1M KOH	250 @ 10 mA/cm^2	42	[30]
Ru-NiCoP/NF	1M KOH	233.77@ 50 mA/cm^2	52.66	[9]
Ti_3C_2Tx/TiO_2/NiFeCo-LDH	1M KOH	155 @ 10 mA/cm^2	98.4	[31]
NiFeCuP@Ni_3S_2	1M KOH	230@ 10 mA/cm^2	42	[32]
p-NFNR@Ni-Co-P	1M KOH	272@10 mA/cm	62	[17]
$Ni_xFe_{1-x}Se_2$-DO	1M KOH	195 @ 10 mA/cm^2	28	[33]
Co_3O_4/Co-Fe oxide	1M KOH	279 @ 10 mA/cm^2	61	[34]
CoP/Ni_2P@HPNCP	1M KOH	294@10 mA/cm	65.5	[22]
a-NiFe-OH/NiFeP	1M KOH	199 @ 10 mA/cm^2	39	[35]
Ni_2CoFe- LDH/N-GO	1M KOH	151 @ 10 mA/cm^2	56.8	[36]
NiFe- LDH/CNT	1M KOH	154 @ 10 mA/cm^2	35	[37]
Carbon-QD/NiFe- LDH	1M KOH	151 @ 10 mA/cm^2	30	[38]
$(Ni,Co)_{0.85}$Se@ NiCo LDH	1M KOH	216 @ 10 mA/cm^2	85	[39]
Ti_2NT_x@MOF-CoP	1M KOH	241@50 mA/cm	96.7	[23]
Mo–NiCoP	1M KOH	269@10 mA/cm	76.7	[24]
CoNi LDH/Ti_3C_2T	1M KOH	257.4@100 mA/cm	68	[40]
NiFeP/MXene	1M KOH	286@50 mA/cm	35	[41]
NiFe LDH/$Ti_3C_2T_x$/NF	1M KOH	200@10 mA/cm	64.2	[42]

Table S3. Comparison of overall water splitting of NiFeCo-LHD@MXene with various electrocatalysts.

Electrocatalyst.	Electrolyte	η (V) @ 10 mA/cm²	Ref
NiFeCo-LHD@MXene	1M KOH	1.41	This work
NiS/Ni	1M KOH	1.64	[3]
np-$(Co_{0.52}Fe_{0.48})_2$ P	1M KOH	1.53	[4]
NiFeCoP/NF	1M KOH	1.56 @ 30 mA/cm²	[8]
Ru-NiCoP/NF	1M KOH	1.50 @ 30 mA/cm²	[9]
CoP/Mo_2CT_x	1M KOH	1.56	[14]
Ni-Co-Sn	1M KOH	1.76@ 50 mA/cm²	[15]
p-NFNR@Ni-Co-P	1M KOH	1.62	[17]
CoP/Ni_2P@HPNCP	1M KOH	1.59	[22]
Ti_2NT_x@MOF-CoP	1M KOH	1.61	[23]
Mo–NiCoP	1M KOH	1.61	[24]
NiFeP/MXene	1M KOH	1.61	[41]

References

1. Zhang, Y.; Gao, L.; Hensen, E.J.M.; Hofmann, J.P. Evaluating the stability of co_2p electrocatalysts in the hydrogen evolution reaction for both acidic and alkaline electrolytes. *ACS Energy Lett.* **2018**, *3*, 1360–1365.
2. Zhang, B.; Xiao, C.; Xie, S.; Liang, J.; Chen, X.; Tang, Y. Iron–nickel nitride nanostructures in situ grown on surface-redox-etching nickel foam: Efficient and ultrasustainable electrocatalysts for overall water splitting. *Chem. Mater.* **2016**, *28*, 6934–6941.
3. Zhu, W.; Yue, X.; Zhang, W.; Yu, S.; Zhang, Y.; Wang, J.; Wang, J. Nickel sulfide microsphere film on ni foam as an efficient bifunctional electrocatalyst for overall water splitting. *Chem. Commun.* **2016**, *52*, 1486–1489.
4. Tan, Y.; Wang, H.; Liu, P.; Shen, Y.; Cheng, C.; Hirata, A.; Fujita, T.; Tang, Z.; Chen, M. Versatile nanoporous bimetallic phosphides towards electrochemical water splitting. *Energy Environ. Sci.* **2016**, *9*, 2257–2261.
5. Djire, A.; Wang, X.; Xiao, C.; Nwamba, O.C.; Mirkin, M.V.; Neale, N.R. Basal plane hydrogen evolution activity from mixed metal nitride mxenes measured by scanning electrochemical microscopy. *Adv. Funct. Mater.* **2020**, *30*, 2001136.
6. Wang, J.; He, P.; Shen, Y.; Dai, L.; Li, Z.; Wu, Y.; An, C. Feni nanoparticles on mo2tic2tx mxene@ nickel foam as robust electrocatalysts for overall water splitting. *Nano Res.* **2021**, *14*, 3474–3481.
7. Wang, W.; Zhao, H.; Du, Y.; Yang, Y.; Li, S.; Yang, B.; Liu, Y.; Wang, L. Rational design and controlled synthesis of v-doped ni3s2/nixpy heterostructured nanosheets for the hydrogen evolution reaction. *Chem.–A Eur. J.* **2021**, *27*, 2463–2468.
8. Cen, J.; Wu, L.; Zeng, Y.; Ali, A.; Zhu, Y.; Shen, P.K. Heterogeneous nifecop/nf nanorods as a bifunctional electrocatalyst for efficient water electrolysis. *ChemCatChem* **2021**, *13*, 4602–4609.
9. Cen, J.; Shen, P.K.; Zeng, Y. Ru doping nicop hetero-nanowires with modulated electronic structure for efficient overall water splitting. *J. Colloid Interface Sci.* **2022**, *610*, 213–220.
10. Xiao, C.; Li, Y.; Lu, X.; Zhao, C. Bifunctional porous nife/nico2o4/ni foam electrodes with triple hierarchy and double synergies for efficient whole cell water splitting. *Adv. Funct. Mater.* **2016**, *26*, 3515–3523.
11. Pang, Y.; Xu, W.; Zhu, S.; Cui, Z.; Liang, Y.; Li, Z.; Wu, S.; Chang, C.; Luo, S. Self-supporting amorphous nanoporous nifecop electrocatalyst for efficient overall water splitting. *J. Mater. Sci. Technol.* **2021**, *82*, 96–104.
12. Ai, Z.; Chang, B.; Xu, C.; Huang, B.; Wu, Y.; Hao, X.; Shao, Y. Interface engineering in the bnns@ ti 3 c 2 intercalation structure for enhanced electrocatalytic hydrogen evolution. *New J. Chem.* **2019**, *43*, 8613–8619.
13. Le, T.A.; Tran, N.Q.; Hong, Y.; Kim, M.; Lee, H. Porosity-engineering of mxene as a support material for a highly efficient electrocatalyst toward overall water splitting. *ChemSusChem* **2020**, *13*, 945–955.
14. Liu, S.; Lin, Z.; Wan, R.; Liu, Y.; Liu, Z.; Zhang, S.; Zhang, X.; Tang, Z.; Lu, X.; Tian, Y. Cobalt phosphide supported by two-dimensional molybdenum carbide (mxene) for the hydrogen evolution reaction, oxygen evolution reaction, and overall water splitting. *J. Mater. Chem. A* **2021**, *9*, 21259–21269.
15. Liu, Y.; Lu, H.; Kou, X. Electrodeposited ni-co-sn alloy as a highly efficient electrocatalyst for water splitting. *Int. J. Hydrogen Energy* **2019**, *44*, 8099–8108.
16. Park, S.; Lee, Y.-L.; Yoon, Y.; Park, S.Y.; Yim, S.; Song, W.; Myung, S.; Lee, K.-S.; Chang, H.; Lee, S.S. Reducing the high hydrogen binding strength of vanadium carbide mxene with atomic pt confinement for high activity toward her. *Appl. Catal. B Environ.* **2022**, *304*, 120989.
17. Feng, Y.; Wang, R.; Dong, P.; Wang, X.; Feng, W.; Chen, J.; Cao, L.; Feng, L.; He, C.; Huang, J. Enhanced electrocatalytic activity of nickel cobalt phosphide nanoparticles anchored on porous n-doped fullerene nanorod for efficient overall water splitting. *ACS Appl. Mater. Interfaces* **2021**, *13*, 48949–48961.
18. Ren, J.; Zong, H.; Sun, Y.; Gong, S.; Feng, Y.; Wang, Z.; Hu, L.; Yu, K.; Zhu, Z. 2d organ-like molybdenum carbide (mxene) coupled with mos 2 nanoflowers enhances the catalytic activity in the hydrogen evolution reaction. *CrystEngComm* **2020**, *22*, 1395–1403.

19. Liu, H.; Cheng, J.; He, W.; Li, Y.; Mao, J.; Zheng, X.; Chen, C.; Cui, C.; Hao, Q. Interfacial electronic modulation of ni3s2 nanosheet arrays decorated with au nanoparticles boosts overall water splitting. *Appl. Catal. B Environ.* **2022**, *304*, 120935.
20. Cui, B.; Hu, B.; Liu, J.; Wang, M.; Song, Y.; Tian, K.; Zhang, Z.; He, L. Solution-plasma-assisted bimetallic oxide alloy nanoparticles of pt and pd embedded within two-dimensional ti3c2t x nanosheets as highly active electrocatalysts for overall water splitting. *ACS Appl. Mater. Interfaces* **2018**, *10*, 23858–23873.
21. Du, C.F.; Sun, X.; Yu, H.; Fang, W.; Jing, Y.; Wang, Y.; Li, S.; Liu, X.; Yan, Q. V4c3t x mxene: A promising active substrate for reactive surface modification and the enhanced electrocatalytic oxygen evolution activity. *InfoMat* **2020**, *2*, 950–959.
22. Zhang, R.; Zhu, R.; Li, Y.; Hui, Z.; Song, Y.; Cheng, Y.; Lu, J. Cop and ni 2 p implanted in a hollow porous n-doped carbon polyhedron for ph universal hydrogen evolution reaction and alkaline overall water splitting. *Nanoscale* **2020**, *12*, 23851–23858.
23. Zong, H.; Qi, R.; Yu, K.; Zhu, Z. Ultrathin ti2ntx mxene-wrapped mof-derived cop frameworks towards hydrogen evolution and water oxidation. *Electrochim. Acta* **2021**, *393*, 139068.
24. Lin, J.; Yan, Y.; Li, C.; Si, X.; Wang, H.; Qi, J.; Cao, J.; Zhong, Z.; Fei, W.; Feng, J. Bifunctional electrocatalysts based on mo-doped nicop nanosheet arrays for overall water splitting. *Nano-Micro Lett.* **2019**, *11*, 55.
25. Shen, B.; Huang, H.; Jiang, Y.; Xue, Y.; He, H. 3D interweaving mxene–graphene network–confined ni–fe layered double hydroxide nanosheets for enhanced hydrogen evolution. *Electrochim. Acta* **2022**, *407*, 139913.
26. Chai, Y.-M.; Shang, X.; Liu, Z.-Z.; Dong, B.; Han, G.-Q.; Gao, W.-K.; Chi, J.-Q.; Yan, K.-L.; Liu, C.-G. Ripple-like nifeco sulfides on nickel foam derived from in-situ sulfurization of precursor oxides as efficient anodes for water oxidation. *Appl. Surf. Sci.* **2018**, *428*, 370–376.
27. Dong, B.; Zhao, X.; Han, G.-Q.; Li, X.; Shang, X.; Liu, Y.-R.; Hu, W.-H.; Chai, Y.-M.; Zhao, H.; Liu, C.-G. Two-step synthesis of binary ni–fe sulfides supported on nickel foam as highly efficient electrocatalysts for the oxygen evolution reaction. *J. Mater. Chem. A* **2016**, *4*, 13499–13508.
28. Yu, L.; Yang, J.F.; Guan, B.Y.; Lu, Y.; Lou, X.W. Hierarchical hollow nanoprisms based on ultrathin ni-fe layered double hydroxide nanosheets with enhanced electrocatalytic activity towards oxygen evolution. *Angew. Chem. Int. Ed.* **2018**, *57*, 172–176.
29. Hao, C.; Wu, Y.; An, Y.; Cui, B.; Lin, J.; Li, X.; Wang, D.; Jiang, M.; Cheng, Z.; Hu, S. Interface-coupling of cofe-ldh on mxene as high-performance oxygen evolution catalyst. *Mater. Today Energy* **2019**, *12*, 453–462.
30. Yu, M.; Zhou, S.; Wang, Z.; Zhao, J.; Qiu, J. Boosting electrocatalytic oxygen evolution by synergistically coupling layered double hydroxide with mxene. *Nano Energy* **2018**, *44*, 181–190.
31. Hao, N.; Wei, Y.; Wang, J.; Wang, Z.; Zhu, Z.; Zhao, S.; Han, M.; Huang, X. In situ hybridization of an mxene/tio 2/nifeco-layered double hydroxide composite for electrochemical and photoelectrochemical oxygen evolution. *RSC Adv.* **2018**, *8*, 20576–20584.
32. Khodabakhshi, M.; Chen, S.; Ye, T.; Wu, H.; Yang, L.; Zhang, W.; Chang, H. Hierarchical highly wrinkled trimetallic nifecu phosphide nanosheets on nanodendrite ni3s2/ni foam as an efficient electrocatalyst for the oxygen evolution reaction. *ACS Appl. Mater. Interfaces* **2020**, *12*, 36268–36276.
33. Xu, X.; Song, F.; Hu, X. A nickel iron diselenide-derived efficient oxygen-evolution catalyst. *Nat. Commun.* **2016**, *7*, 12324.
34. Wang, X.; Yu, L.; Guan, B.Y.; Song, S.; Lou, X.W. Metal–organic framework hybrid-assisted formation of co3o4/co-fe oxide double-shelled nanoboxes for enhanced oxygen evolution. *Adv. Mater.* **2018**, *30*, 1801211.
35. Liang, H.; Gandi, A.N.; Xia, C.; Hedhili, M.N.; Anjum, D.H.; Schwingenschlogl, U.; Alshareef, H.N. Amorphous nife-oh/nifep electrocatalyst fabricated at low temperature for water oxidation applications. *ACS Energy Lett.* **2017**, *2*, 1035–1042.
36. Zhou, D.; Cai, Z.; Lei, X.; Tian, W.; Bi, Y.; Jia, Y.; Han, N.; Gao, T.; Zhang, Q.; Kuang, Y. Nicofe-layered double hydroxides/n-doped graphene oxide array colloid composite as an efficient bifunctional catalyst for oxygen electrocatalytic reactions. *Adv. Energy Mater.* **2018**, *8*, 1701905.
37. Gong, M.; Li, Y.; Wang, H.; Liang, Y.; Wu, J.Z.; Zhou, J.; Wang, J.; Regier, T.; Wei, F.; Dai, H. An advanced ni–fe layered double hydroxide electrocatalyst for water oxidation. *J. Am. Chem. Soc.* **2013**, *135*, 8452–8455.
38. Tang, D.; Liu, J.; Wu, X.; Liu, R.; Han, X.; Han, Y.; Huang, H.; Liu, Y.; Kang, Z. Carbon quantum dot/nife layered double-hydroxide composite as a highly efficient electrocatalyst for water oxidation. *ACS Appl. Mater. Interfaces* **2014**, *6*, 7918–7925.
39. Xia, C.; Jiang, Q.; Zhao, C.; Hedhili, M.N.; Alshareef, H.N. Selenide-based electrocatalysts and scaffolds for water oxidation applications. *Adv. Mater.* **2016**, *28*, 77–85.
40. Hu, L.; Li, M.; Wei, X.; Wang, H.; Wu, Y.; Wen, J.; Gu, W.; Zhu, C. Modulating interfacial electronic structure of coni ldh nanosheets with ti3c2tx mxene for enhancing water oxidation catalysis. *Chem. Eng. J.* **2020**, *398*, 125605.
41. Chen, J.; Long, Q.; Xiao, K.; Ouyang, T.; Li, N.; Ye, S.; Liu, Z.-Q. Vertically-interlaced nifep/mxene electrocatalyst with tunable electronic structure for high-efficiency oxygen evolution reaction. *Sci. Bull.* **2021**, *66*, 1063–1072.
42. Li, Z.; Wang, X.; Ren, J.; Wang, H. Nife ldh/ti3c2tx/nickel foam as a binder-free electrode with enhanced oxygen evolution reaction performance. *Int. J. Hydrogen Energy* **2022**, *47*, 3886–3892.
